# Sibiriline, a novel dual inhibitor of necroptosis and ferroptosis, prevents RIPK1 kinase activity and (phospho)lipid peroxidation as a potential therapeutic strategy

**DOI:** 10.1038/s41420-025-02852-8

**Published:** 2025-11-28

**Authors:** Claire Delehouzé, Melodie Mallais, Arnaud Comte, Romain Lucas, Blandine Baratte, Sophie Bélal, Axelle Autret, Nathalie Py, Rémy Steinschneider, Lucie Adoux, Benjamin Saintpierre, Franck Letourneur, Thomas Robert, Céline Cougoule, Caio Bomfim, Rémi Planès, David Péricat, Jeannette Chloë Bulinski, Marie-Thérèse Dimanche-Boitrel, Peter Goekjian, Etienne Meunier, Morgane Rousselot, Derek A. Pratt, Stéphane Bach

**Affiliations:** 1 SeaBeLife Biotech, Place Georges Teissier, 29680 Roscoff, France; 2 Sorbonne Université, CNRS, Laboratoire de Biologie Intégrative des Modèles Marins, LBI2M, F-29680 Roscoff, France; 3 Department of Chemistry and Biomolecular Sciences, University of Ottawa, Ottawa, ON Canada; 4 Université de Lyon, CNRS UMR 5246, ICBMS, Chimiothèque, Université Claude Bernard Lyon 1, F-69622 Villeurbanne, France; 5 Sorbonne Université, CNRS, Kinase Inhibitor Specialized Screening facility, KISSf, F-29680 Roscoff, France; 6 Neuron Experts, Cité de la cosmétique, 2 rue Odette Jasse, 13015 Marseille, France; 7 Plateforme GenomIC, Université de Paris, Institut Cochin, INSERM-CNRS, F-75014 Paris, France; 8 Institut de Pharmacologie et Biologie Structurale (IPBS), Université de Toulouse, CNRS, Toulouse, France; 9 France Univ Rennes, Inserm, EHESP, Irset (Institut de Recherche en santé, environnement et travail) - UMR_S 1085, F-35000 Rennes, France; 10 Université de Lyon, CNRS UMR 5246, ICBMS, Laboratoire Chimie Organique 2-Glycosciences, Université Claude Bernard Lyon 1, F-69622 Villeurbanne, France

**Keywords:** Mechanism of action, Drug discovery

## Abstract

In the past two decades, various non-apoptotic pathways of regulated cell death have been identified; a small subset of these, including necroptosis and ferroptosis, manifests the phenotypic features of necrotic death. These two regulated necroses are being extensively studied because of their putative roles in severe acute and chronic pathologies. Moreover, as these regulated necrotic pathways are coactivated in a number of common pathologies, the development of multi-target directed ligands (that is, the use of a polypharmacological strategy) is a path-breaking avenue of research. In this study, we determined that the 7-azaindole derivative, sibiriline, inhibited both RIPK1-driven necroptosis (induced by Tumor Necrosis Factor-α) and ferroptosis (triggered by various classes of ferroptosis inducers), with EC_50_s against each in the µM range. We next performed a combined large-scale transcriptomic study in order to determine the molecular mechanisms of action of sibiriline. We identified the stress response protein heme oxygenase-1 (HMOX1) as the main biomarker of ferroptosis inhibition by sibiriline. We hypothesized that this compound reacts as an antioxidant to block ferroptosis; indeed, we found that sibiriline inhibits lipid peroxidation by trapping phospholipid-derived peroxyl radicals as a radical-trapping antioxidant (RTA). Taken together, these results show that sibiriline is a new dual inhibitor of necroptosis and ferroptosis cell death pathways; it works by inhibition of both RIPK1 kinase and (phospho)lipid peroxidation. We also demonstrate the in vitro efficacy of sibiriline to inhibit cell death in cell-based models of Parkinson’s disease and cystic fibrosis. These findings shed light on the high therapeutic potency of RIPK1 inhibitors with RTA activity.

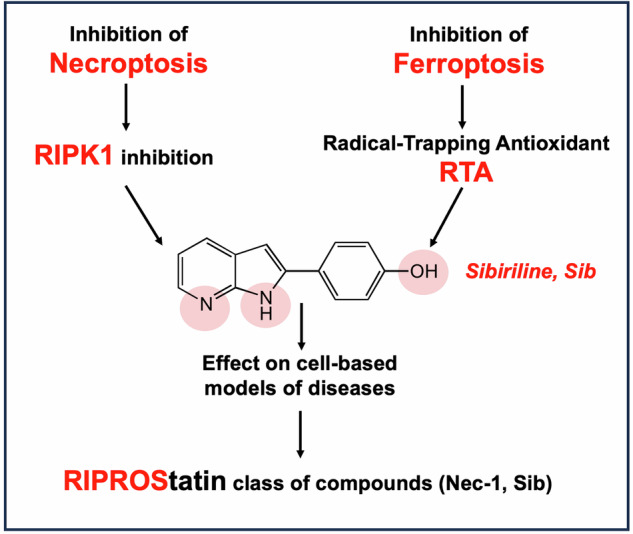

## Introduction

**Fig. 1:**
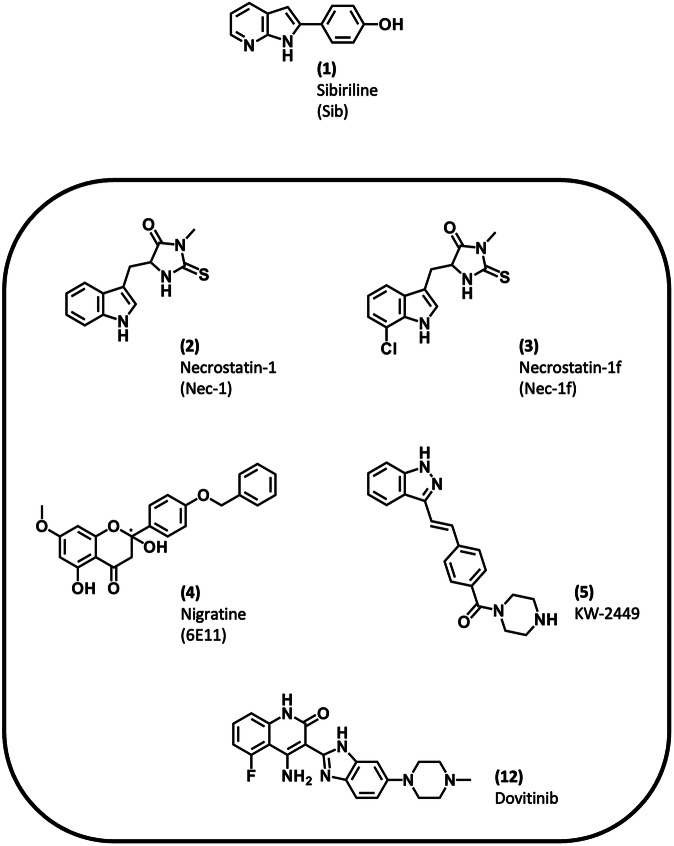
Structures of sibiriline (Sib, 1) and previously reported dual necroptosis/ferroptosis inhibitors, necrostatin-1 (Nec-1, 2) [24, 50], necrostatin-1f (Nec-1f, 3) [26], nigratine (6E11, 4) [25], KW-2449 (5) [27] and Dovitinib (12) [28].

Over the past decade, significant progress has been made in the study of necrotic death pathways. The Nomenclature Committee on Cell Death has identified seven regulated necrotic cell death pathways, including notably necroptosis and ferroptosis [1]. The first inhibitor of necroptosis described in the literature was necrostatin-1, an inhibitor of the receptor-interacting protein kinase 1 (RIPK1) [2]. Since then, RIPK1 has emerged as a crucial component of the inflammatory response [3, 4]. Dysregulation of RIPK1 signaling has been implicated in driving the pathology of several diseases, including cancer, autoimmune diseases, and multiple aging-related diseases [5, 6]. Consequently, RIPK1 is an attractive therapeutic target and several clinical trials are currently underway to investigate the efficacy of RIPK1 inhibitors, after successfully completing the safety clinical phase, in patients with rheumatoid arthritis, acute graft-versus-host disease, cardiac surgery-associated acute kidney injury, or ulcerative colitis ([7] and data collected from clinicaltrials.gov). Among the kinase inhibitors currently under investigation, sibiriline, also known as 4-(1H-pyrrolo[2,3-b]pyridin-2-yl)phenol (Fig. 1), has demonstrated: (i) a potent inhibition of RIPK1; (ii) only a very low cytotoxic effect on human peripheral blood leukocytes, as well as on human retinal pigment epithelial cells; and (iii) has shown promising results in protecting liver tissue in an in vivo model of autoimmune hepatitis [8]. Sib is thus considered as a promising treatment option for RIPK1-associated liver disorders. In addition, Sib has a favorable toxicology profile for clinical use [9].

Ferroptosis is an iron-dependent, non-apoptotic mode of cell death characterized by the accumulation of (phospho)lipid hydroperoxides (pLOOH). Ferrostatin-1 (Fer-1) and liproxstatin-1 (Lip-1), the first potent inhibitors of ferroptosis, were reported by the Stockwell and Conrad groups, respectively [10, 11]. They were subsequently demonstrated to be radical-trapping antioxidants (RTAs), which inhibit (phospho)lipid peroxidation by reacting with the radicals that propagate the radical chain reaction [12]. This class of ferroptosis inhibitors holds promise for the development of innovative therapeutic agents [13–17].

To date, no molecule targeting necroptosis or ferroptosis has reached the market. As already described in the literature, many effective drugs act via modulation of multiple proteins or pathways rather than single targets [18]. Such an approach, using single pharmaceutical agents acting on multiple pathways, can be applied to regulated necrosis-related diseases. Indeed, numerous studies have provided evidence that complex diseases involve the activation of multiple cell death pathways [19]. Furthermore, it has been established that there are interactions among regulated cell death pathways [20]. The crosstalk between these signaling pathways in human disease is currently being investigated, and some studies suggest synergistic effects in pathologies in which two or more cell death pathways are involved. This has been demonstrated for necroptosis and ferroptosis in acute kidney failure [21] or in ischemic stroke [22]. Therefore, developing compounds that target multiple cell death pathways simultaneously could be a promising therapeutic approach for treating patients, especially for those diseases that currently lack adequate therapeutic options. Currently, five molecules have been described that exhibit dual inhibitory effects on necroptosis and ferroptosis pathways. These molecules, presented in Fig. 1, were shown to inhibit necroptosis by targeting RIPK1, and their efficacy in inhibiting ferroptosis has been demonstrated through cell-based phenotypic approaches [11, 23–28].

Here, we show that Sib suppresses cell death induced by several classes of ferroptosis inducers. Utilizing the Fluorescence ENabled Inhibited autoXidation (FENIX) cell-free assay, the RTA activity of Sib was unambiguously demonstrated, and from structure-reactivity relationships was shown to derive largely from reaction of the phenolic moiety with (phospho)lipidperoxyl radicals. Taken together, our results open the door to the use of dual RTA and RIPK1 inhibitors, a class of drugs that we propose to name RIPROStatins (for RIPK1 inhibitors that have reactive oxygen species (ROS) scavenging activity), as innovative therapies for complex human diseases.

## Results

### Sibiriline, a necroptosis inhibitor, inhibits ferroptotic cell death

Sib selectivity was previously assessed against 456 kinases using a competition binding assay (KINOMEscan; DiscoverX, San Diego, CA, USA) [8], identifying RIPK1 as the main target (K_d_ = 218 nM), with an IC_50_ value (i.e., the concentration of drug at which 50% of the kinase activity is inhibited) of 1.1 µM confirmed by enzymatic assay (Fig. S1A). In this study, IC_50_ values were determined using ATP concentrations corresponding to the K_M_ values for ATP (K_M, ATP_) for each kinase (except EPHB6, technical problems precluded its use). As Sib is an ATP-competitive inhibitor, IC_50_ values were adjusted using the Cheng-Prusoff equation (Fig. S1B). All kinases tested showed inhibition in the µM range. Theoretical IC_50_ values at 2.5 mM ATP—approximating cellular ATP levels—were also calculated. Among the tested kinases, RIPK1 was predicted to be the most potently inhibited under physiological conditions, due to its high K_M,ATP_.

**Fig. 2:**
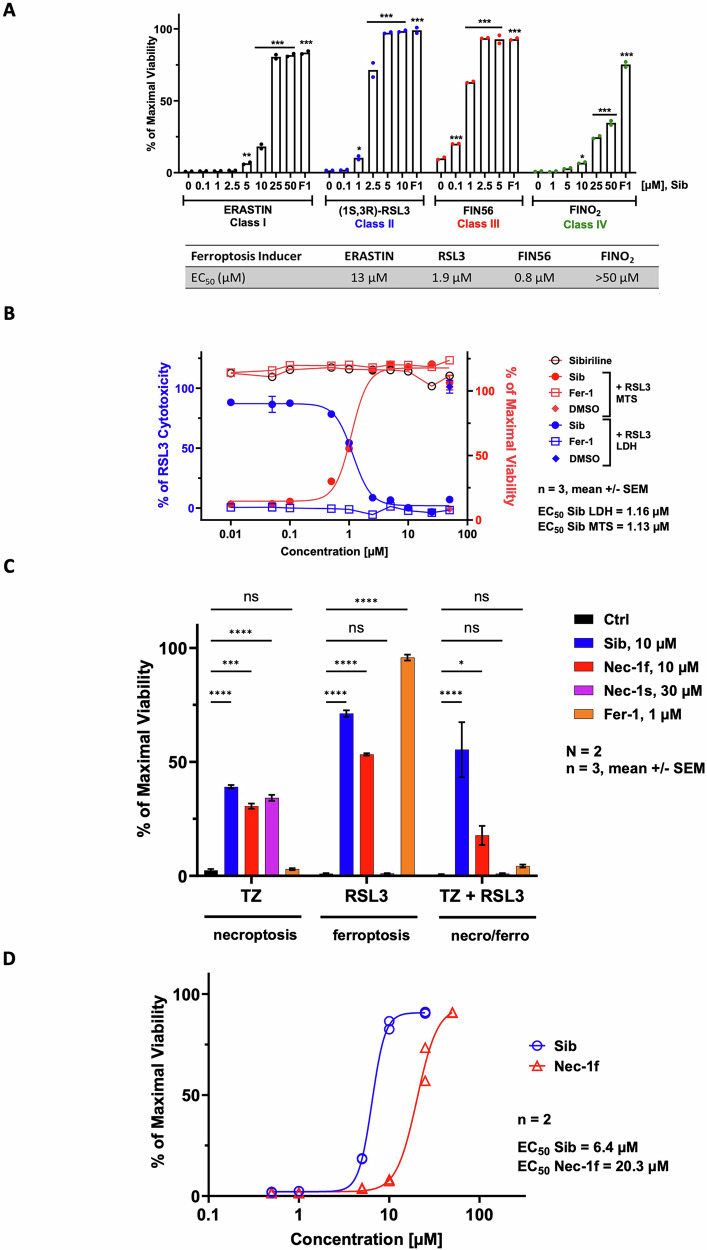
Sibiriline (1) is an inhibitor of ferroptosis cell death. **A** SH-SY5Y cells were treated with either 10 µM Erastin, 5 µM RSL3, 10 µM FIN56 or 25 µM FINO_2_, and increasing concentrations of Sib up to 50 µM or until reaching maximal cellular viability or 1 µM of ferrostatin-1 (Fer-1/F1) as a control. Cell viability was determined after 24 h of treatment, using the MTS assay. The bar graph represents the mean of two replicates. **B** SH-SY5Y cells were co-treated for 24 h with 5 µM RSL3 and increasing concentrations of Sib or Fer-1. Cell death was estimated by the lactate dehydrogenase (LDH) release assay. Results are plotted in % of LDH release measured in cells treated with RSL3 alone (left axis, colored blue). Cell viability was evaluated by MTS reduction assay. Results obtained (colored red) were plotted as % of maximal viability with DMSO-treated cells (right axis). Data are shown as the mean +/- SEM of three replicates. **C** NIH3T3 cells were treated with either 1 µM RSL3, 5 ng/ml TNFα and 20 µM z-VAD.fmk (TZ) or a combination of both treatment (TZ + RSL3) and 10 µM of Sib or Nec-1f, 30 µM Nec-1s or 1 µM of Fer-1. Cell viability was evaluated by MTS reduction assay after 16 h of treatment. Data are shown as the mean ± SEM of three replicates of two independent experiments. **D** NIH3T3 cells were treated with 5 ng/ml TNFα, 20 µM z-VAD.fmk and 1 µM RSL3 and increasing concentrations of Sib or Nec-1f. Cell viability was determined after 16 h of treatment using an MTS assay. Data are shown as the mean ± SEM of two replicates. EC_50_ values were calculated using graphpad prism software. Statistical analysis was performed using two-way ANOVA and Tukey’s multiple comparisons test using graphpad prism software. *p < 0.05, **p < 0.01, ***p < 0.001 and ****p < 0.0001 vs controls.

Given previous findings that the flavanone nigratine inhibits both necroptosis and ferroptosis [25], we evaluated the effect of Sib on ferroptotic cell death. SH-SY5Y cells were treated with four ferroptosis inducers (FINs), and Sib showed dose-dependent protection against all, particularly RSL3 (Fig. 2A), which was used in subsequent assays. LDH release assays confirmed that Sib reduced RSL3-induced necrotic cell death in a dose-dependent manner (Fig. 2B). Sib also protected HT22 cells from RSL3-induced ferroptosis (Suppl. Fig. S2). An inactive indolyl analogue of Sib, Sib-i, previously shown to be ineffective in blocking necroptosis [8], interestingly protected SH-SY5Y cells from ferroptosis (EC_50_ = 0.09 µM; Suppl. Fig. S3). It was thus renamed Sib-f to reflect its ferroptosis-inhibitory activity.

We next used NIH3T3 cells as a model to investigate the effect of Sib on dual induction of necroptosis and ferroptosis. As described by Tonnus et al. [26] the cells may undergo both necroptosis induced by TNFα and z-VAD.fmk (TZ), and ferroptosis induced by RSL3. We thus induced either necroptosis, ferroptosis or both necroptosis and ferroptosis simultaneously in NIH3T3 cells. Treatment with Sib at 10 µM maintained cellular viability under all three conditions. This dual inhibition of ferroptosis and necroptosis was previously observed by Tonnus et al. [26] for Nec-1f, but not for Nec-1s or Fer-1, which can only protect cells from necroptosis or ferroptosis, respectively. These results were confirmed by the data obtained in this study and reported in Fig. 2C. In order to compare the efficacy of Sib with that of Nec-1f in this model of both necroptosis and ferroptosis, NIH3T3 cells were then treated with TZR cocktail (TNFα + z-VAD + RSL3). Sib showed dose-dependent inhibition of this dual cell death induction pattern, exhibiting better efficacy than Nec-1f (Fig. 2D).

**Fig. 3:**
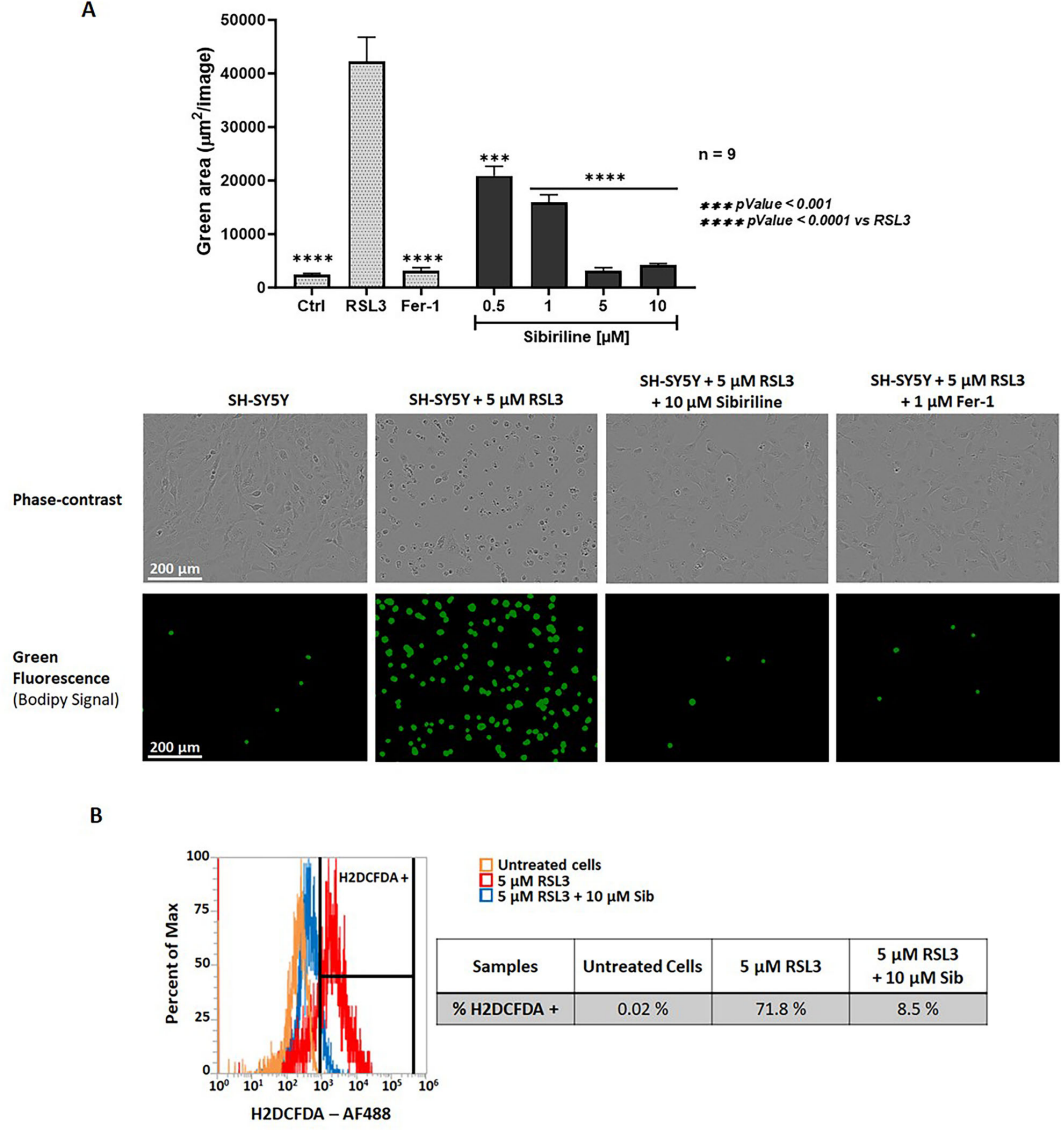
Sibiriline (1) reduces hallmarks of ferroptosis cell death. **A** Lipid peroxidation was detected by cellular BODIPY 581/591 C11 staining. Fluorescence was recorded with the IncuCyte S3 live cell imaging apparatus. Data are shown as the mean ± SEM of nine replicates. Representative phase-contrast and fluorescence images of SH-SY5Y cells stained with BODIPY 581/591 C11 probe, visualized using the IncuCyte S3 live-cell imaging system. **B** SH-SY5Y cells treated with DMSO or with 5 µM RSL3 with or without 10 µM Sib were stained with H2DCFDA dye. H2DCFDA fluorescence signal, indicative of intracellular ROS level, was measured in the Alexa Fluor 488 channel (AF488) using an Attune^TM^ NxT flow cytometer. Representative histograms depicting the ROS production in RSL3-treated SH-SY5Y cells (red), Sib + RSL3-treated cells (blue) or vehicle only (DMSO) cells (orange). The analysis was performed with 10^5^ cells for each condition and the percentage were calculated with Attune^TM^ NxT software (Thermo Fisher).

The hallmarks of ferroptotic cell death include increased (phospho)lipid peroxidation associated with an elevation of intracellular ROS [1]. To investigate the effects of sibiriline on ferroptosis we used the C11-BODIPY 581/591 probe as a sensor of (phospho)lipid peroxidation (Fig. 3A) and the 2’,7’-Dichlorodihydrofluorescein diacetate (H_2_DCFDA) dye as an intracellular ROS probe (Fig. 3B) on SH-SY5Y cells. As expected, RSL3 induced high levels of (phospho)lipid peroxidation. Treatment with 10 µM of sibiriline decreased (phospho)lipid peroxidation to the level of untreated control cells (Fig. 3A). As displayed in Fig. 3B, the deleterious increase in intracellular ROS induced by RSL3 was almost completely abrogated by treatment with Sib.

Taken together, these results demonstrate that Sib acts as an inhibitor of ferroptotic cell death as well as a necroptosis inhibitor and can inhibit both pathways when they are activated simultaneously inside the cell.

### Sibiriline modulates expression of ferroptosis-related genes in a ferroptosis cell death model

**Fig. 4:**
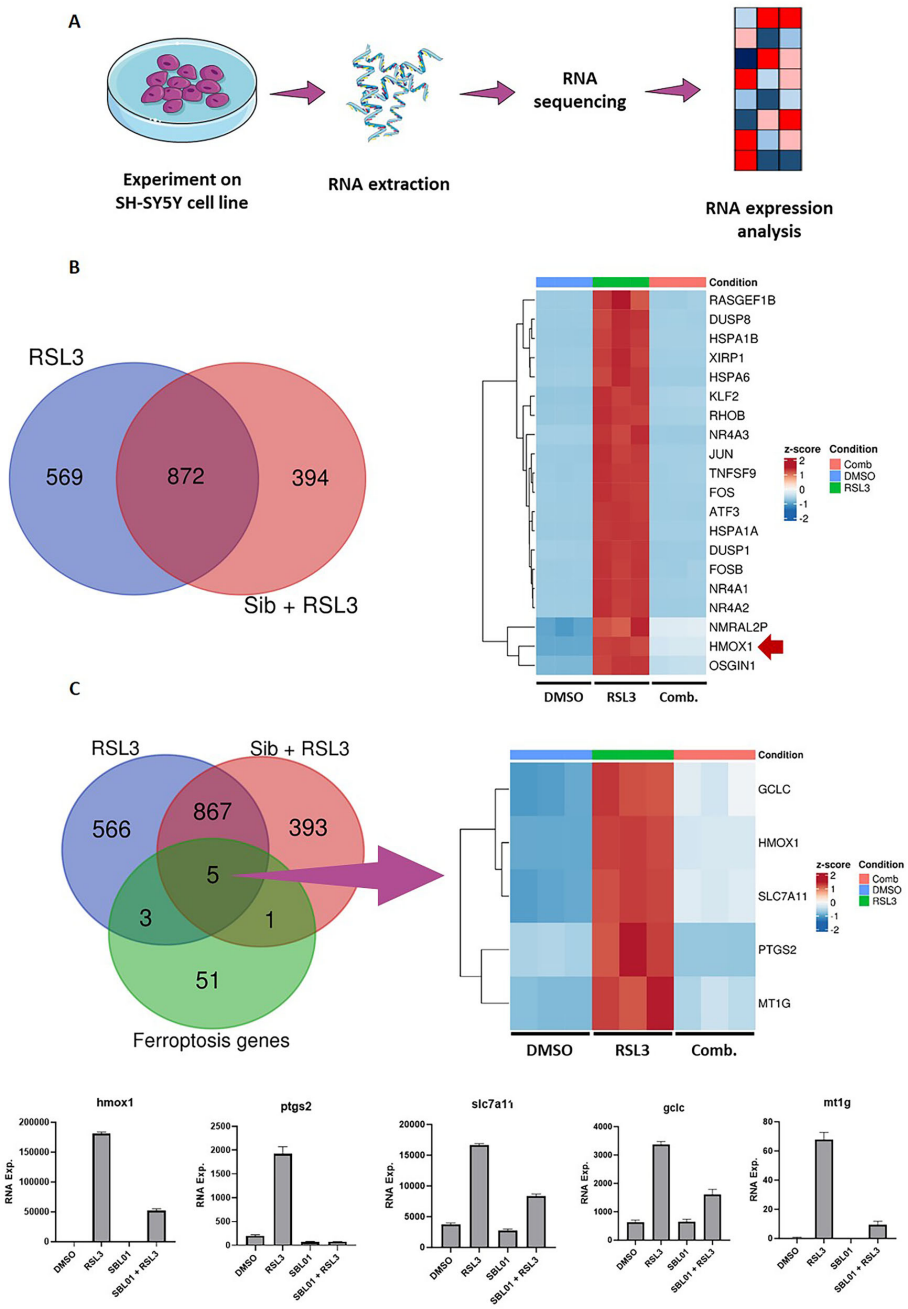
Sibiriline (1) modulates transcription of ferroptosis-related genes. **A** Simplified representation of the workflow used for the transcriptomic-based study. **B** Overlapping of differentially expressed genes (DEGs) in SH-SY5Y cells treated with 5 µM RSL3 vs DMSO (labeled as RSL3, blue circle) and cells treated with RSL3 + Sib (20 µM) vs RSL3 (labeled as Sib + RSL3, red circle). Heatmap showing the 20 most RSL3-DEG genes in RSL3, Sib+RSL3 (Comb) or DMSO treated cells. **C** Overlapping of DEGs in RSL3 and Sib+RSL3 treated cells with 60 ferroptosis-related genes. The heatmap shows the mRNA expression profile of the five selected genes in RSL3, Sib+RSL3 (Comb) or DMSO treated cells. The lower panel shows the representative histograms of mRNA expression for each of the five genes in each experimental condition.

To investigate Sib’s mechanism of action, we performed RNAseq analysis to assess transcriptional responses to chemical treatments, focusing on early ferroptosis induced by RSL3 (Fig. 4A). Differentially expressed genes (DEGs) were identified using a p-value < 0.05 and fold change > 2. A Venn diagram compared DEGs from RSL3-treated cells and Sib+RSL3 co-treated cells (Fig. 4B). Among 1441 and 1266 DEGs respectively, 872 were common. A heatmap of the top 20 DEGs showed that Sib reversed RSL3-induced transcriptional changes, restoring gene expression toward control levels. DEGs also intersected with a published list of 60 ferroptosis-related genes [29] (Fig. 4C), revealing five shared genes (HMOX1, PTGS2, SLC7A11, GCLC, MT1G), represented in a heatmap and bar plots. HMOX1 expression was most affected, being upregulated by RSL3 and suppressed by Sib.

### Sibiriline acts as an RTA to inhibit ferroptosis

**Fig. 5:**
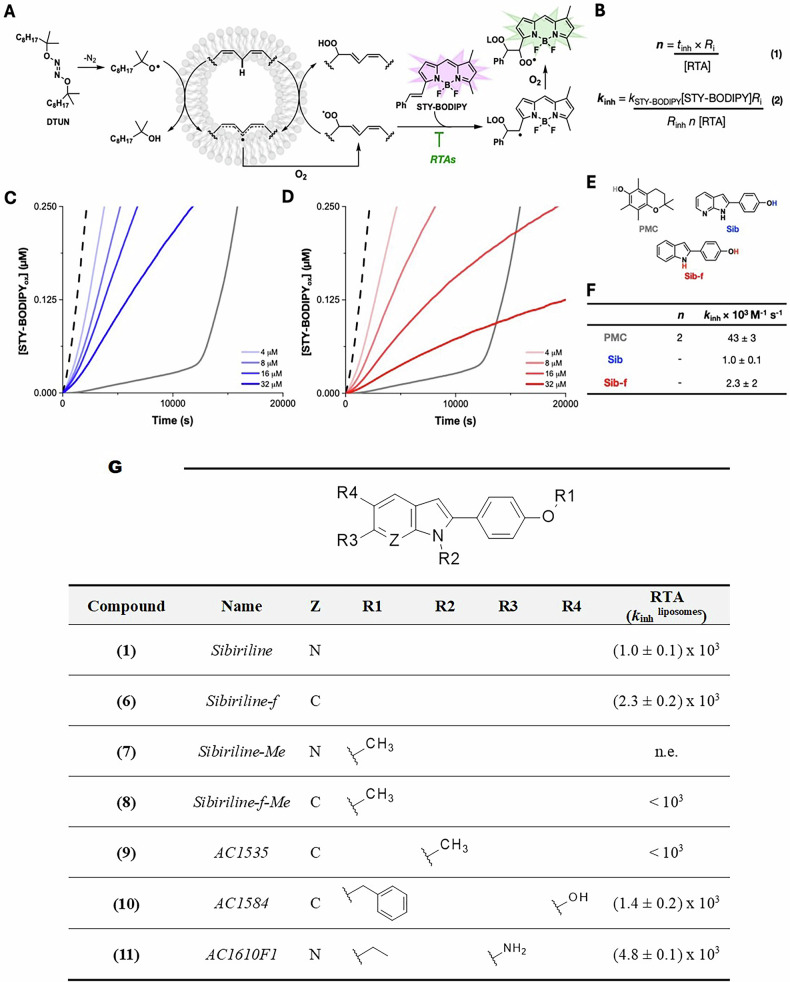
Sib (1) and Sib-f (6) suppress phospholipid peroxidation via radical-trapping antioxidant (RTA) activity. **A** The FENIX assay is based upon the DTUN-initiated co-autoxidation of membrane phospholipids and STY-BODIPY, which is used as a signal carrier to enable reaction monitoring by fluorescence. **B** Radical-trapping stoichiometry (*n*) and apparent inhibition rate constant (*k*_inh_) can be determined directly from reaction progress data from eqs 1 and 2. **C** Sibiriline (**1**) and (**D**) sibiriline-f (**6**) inhibited FENIX co-autoxidations. Phosphatidylcholine liposomes (1 mM) in the presence of STY-BODIPY (1 µM) initiated with DTUN (0.2 mM) inhibited with 4-32 µM of Sib (**1**) or Sib-f (**6**). **E** Chemical structures of RTAs used in the FENIX assay. Quintessential phenolic RTA, 2,2,5,7,8-pentamethychroman-1-ol (PMC) (gray), used in the determination of the rate of initiation (*R*_*i*_), sibiriline (blue) and sibiriline-f (red). **F** Summary of apparent inhibition rate constants (*k*_inh_) and radical-trapping stoichiometry (*n*) derived using eqs 1 and 2. **G** Evaluation of lipid peroxidation-inhibiting activity of sibiriline (**1**) and its derivatives by FENIX assay. The RTA effect is expressed in *k*_inh_^liposomes^ (see [32] for details). Values reported in the table were determined from the curves reported in Fig S4. n.e.: no significant effect detected up to 32 µM.

Phenols comprise the archetype radical-trapping antioxidant scaffold [30] and indole-containing drugs, such as 15-LOX-1 inhibitor PD146176, have off-target RTA activity [31]. As the Sib scaffold harbors a phenol group and an indole or azaindole, we next evaluated whether sibiriline acts as an RTA to suppress ferroptosis initiated by various classes of chemical inducers. Considering that the DPPH assay results do not correlate with efficacy of ferroptosis inhibition [32], we used FENIX assay to evaluate the RTA activity of Sib and Sib-f against (phospho)lipid-derived peroxyl radicals. The FENIX assay provides the key quantitative measure of RTA activity—the apparent inhibition rate constant (*k*_inh_)—which was shown to correlate with potency at suppressing RSL3-induced ferroptosis (in mouse embryonic fibroblasts) [32]. As illustrated in Fig. 5A, the FENIX assay monitors liposomal (phospho)lipid peroxidation fluorometrically, by the competitive co-autoxidation of STY-BODIPY and membrane phospholipids. The assay utilizes a lipophilic hyponitrite initiator (di-*tert*-undecyl hyponitrite, DTUN) to generate a constant supply of initiating alkoxyl radicals over a convenient timescale, enabling the derivation of *k*_inh_ and the radical-trapping stoichiometry (*n*) according to eqs 1 and 2 in Fig. 5B. As shown in Fig. 5C and Fig. 5D, both Sib and Sib-f suppressed STY-BODIPY oxidation—and therefore (phospho)lipid peroxidation—with *k*_inh_ = (1.0 ± 0.1) × 10^3^ and (2.3 ± 0.2) × 10^3 ^M^−1^ s^−1^, respectively. The lack of a distinct inhibited period in Sib and Sib-f inhibited co-autoxidations precluded the determination of *n*, and as such, it was assumed to be one for the derivation of *k*_inh_. Sib-f was approximately two-fold more reactive against phospholipid-derived peroxyls compared to Sib, consistent with the absence of the electronegative ring nitrogen found in Sib, which is known to slow HAT in both phenolic and aminic RTAs [33].

### Structure-activity relationship study of RTA activity

The presence of both phenolic and aminic RTA moieties in Sib and Sib-f prompted us to modify the Sib scaffolds to probe which of each were more important for RTA activity (Fig. 5G and S4). When the free phenol of Sib (**1**) was methylated to give Sib-Me (**7**), no significant effect on phospholipid peroxidation (pLPO) could be observed by the FENIX assay at concentrations as high as 32 µM, indicating the phenol of Sib is critical to its RTA activity and further suggesting the indole N-H of Sib is unreactive towards phospholipid-derived peroxyls. Likewise, only a very modest retardation of pLPO was observed when either the phenol or indole of Sib-f (**6**) was methylated to give Sib-f-Me (**8**) or AC1535 (**9**), respectively, corresponding to *k*_inh_ < 10^3 ^M^-1^s^-1^ for each. These results suggest that the indole N-H in Sib-f retains some RTA activity in the absence of the pyridine nitrogen found in Sib, but is very modest. We surmised the addition of a phenolic moiety para to the indole N-H in Sib-f would result in an enhancement in trapping of phospholipid-derived peroxyl radicals as the electron-donating character of the indole should weaken the O-H bond dissociation enthalpy as well as reduce the hydrogen-bond acidity of the phenol [34]. Indeed, AC1584 (**10**) was similarly effective at trapping phospholipid-derived peroxyls (*k*_inh_ = (1.4 ± 0.2) × 10^3 ^M^-1^s^-1^). Alternatively, adding an amine group to Sib as in AC1610F1 (**11**) was expected to compensate for the electron-withdrawing nature of the pyridine nitrogen. Indeed, AC1610F1 was the most reactive Sib derivative assayed in our structure-reactivity study and reacted with phospholipid-derived peroxyl radicals over 2-fold faster than Sib-f (*k*_inh_ = (4.8 ± 0.1) × 10^3 ^M^−1^ s^−1^), the second most reactive derivative tested in FENIX. Thus, not only does the amine compensate for the electron-withdrawing nature of the pyridine nitrogen, but seemingly further increases electron-density at the indole nitrogen, enabling H-atom transfer. We also analyzed the reactivity of this set of compounds using the DPPH assay. The data obtained are reported in Table S1. As expected from previous reports, we found the DPPH assay to be a poor predictor of lipophilic RTA activity as observed using the FENIX assay.

We hypothesized the enhanced reactivity of AC1610F1 (**11**) in FENIX would translate to potent ferroptosis suppression in cells. As such, we next compared the activity of the most potent RTA derivative, AC1610F1 (**11**), to Sib, in cell-based assays of ferroptosis. Mouse embryonic fibroblasts NIH3T3, human fibrosarcoma cells HT1080, human neuroblastoma SH-SY5Y, mouse embryonic fibroblasts Pfa1, and mouse hippocampal neuronal HT22 cells were treated with a covalent inhibitor of GPx4 (RSL3) to induce ferroptosis. The results are reported in Table 1. Consistent with reports by Shah et al. [32], the FENIX assay accurately predicted inhibition of ferroptosis, as the best lipophilic RTA in FENIX, AC1610F1 (**11**), was more potent in all cell lines tested, with EC_50_ in the sub-micromolar range (from EC_50_ = 0.065 to 0.74 µM).

**Table 1:** Activity of sibiriline (1) and its derivative AC1610F1 (11) using FENIX assay and ferroptosis model cell-based assays.

Molecule	RTA (k_inh liposomes_)	EC_50_^SHSY-5Y^ (5 µM RSL3)	EC_50_^HT22^ (1 µM RSL3)	EC_50_^HT1080^ (1 µM RSL3)	EC_50_^pfa1^ (1 µM RSL3)	EC_50_^NIH3T3^ (1 µM RSL3)
**(1) Sibiriline**	(1.0 ± 0.1) x 10^3^	(1.0 ± 0.2) µM	(23.9 ± 1.5) µM	(11.1 ± 1.1) µM	(7.6 ± 2.8) µM	(6.05 ± 0.4) µM
**(11) AC1610F1**	(4.8 ± 0.1) x 10^3^	(64.7 ± 7.3) nM	(0.72 ± 0.09) µM	(0.13 ± 0.01) µM	(0.49 ± 0.06) µM	(0.48 ± 0.3) µM

SH-SY5Y, HT22, HT1080, Pfa1 and NIH3T3 cells were co-treated 24 h with RSL3 (at 5 µM for SHSY-5Y and 1 µM for the other cell lines) and increasing concentrations of Sib (**1**) or AC1610F1 (**11**). EC_50_ (mean ± SEM) were determined using GraphPad PRISM software from the dose-response curves reported on Fig S5 and Fig S6 for Sib and AC1610F1, respectively.

### Effect of sibiriline on the induction of ferroptosis in cystic fibrosis patient cells

**Fig. 6:**
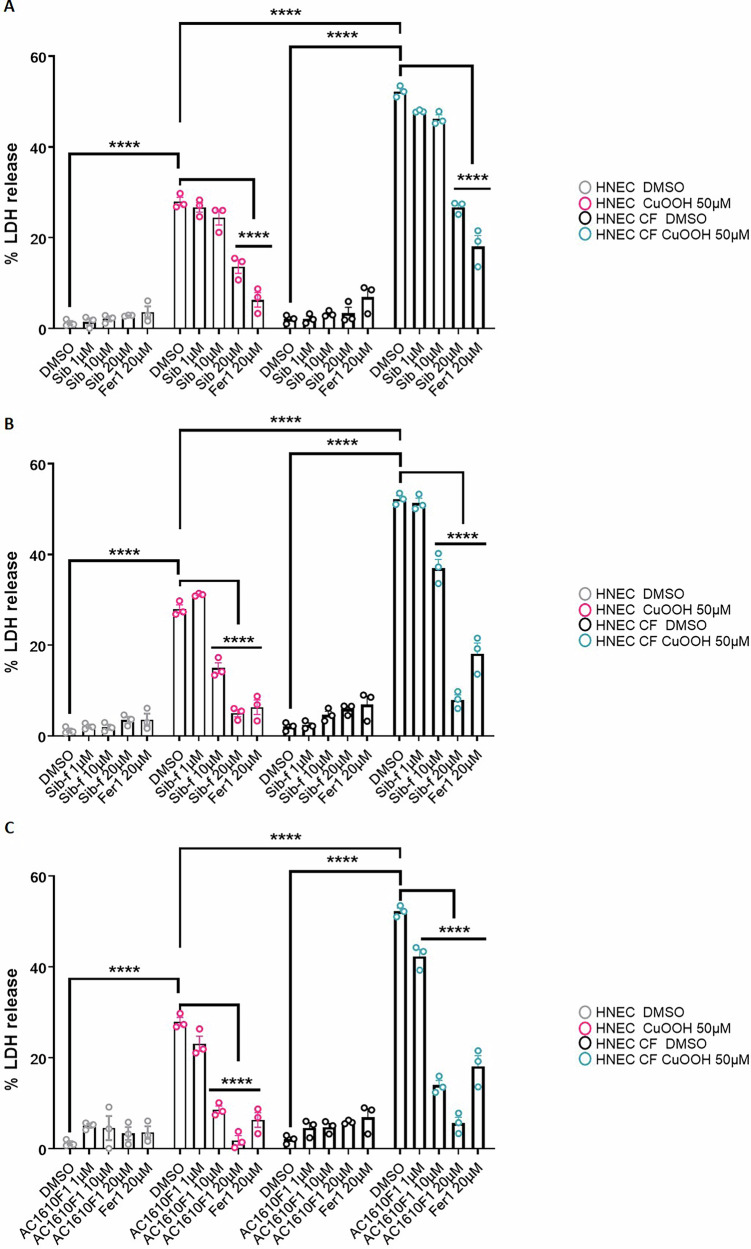
Efficacy of the identified compounds against Cumen Hydroperoxide-induced cell death in healthy or Cystic Fibrosis (CF) nasal epithelial cells. **A**–**C**. LDH release in primary Human Nasal Epithelial Cells (HNECs) from healthy or cystic fibrosis patients exposed to 50 µM of Cumene Hydroperoxide (CuOOH, 50 µM, 8 h) in presence/absence of various concentration of inhibitory compounds. Here Fer1 stands for ferrostatin-1 and was used as internal inhibitory control of CuOOH-induced cell death. The molecules sibiriline (Sib) (**1**), sibiriline-f (Sib-f) (**6**) and AC1610F1 (**11**) are described along the manuscript. ****P ≤ 0.0001, 2-way Anova with multiple comparisons. Values are expressed as mean ± SEM from one experiment (in triplicate) from one independent donor (d1, CF) performed at least three times.

Next, we investigated our compounds in cell-based models of ferroptosis-related disease. Recent studies have suggested that cells from cystic fibrosis (CF) patients exhibit strong sensitivity to ferroptosis induction. Due to defective mucus production and clearance, the airways of CF patients favor chronic and life-threatening bacterial and fungal infections. In collaboration with the Hospital of Toulouse (France), we used previously described nasal brushes obtained from one healthy and one CF patient carrying the ∆F508 mutation in the CF transmembrane conductance regulator for these experiments (CFTR; referred to in the text and figure legends as Healthy and CF donors 1; Fig. 6) [35]. We measured the ability of cells from each to undergo lysis upon exposure to cumene hydroperoxide (CuOOH), a well-known and potent inducer of ferroptosis [36]. LDH release assays showed that CuOOH-driven cell lysis was exacerbated in CF nasal epithelial cells, a process that was reduced by the use of ferrostatin-1, a well-known ferroptosis inhibitor (Fig. 6A–C). Further studies showed that Sib-f (**6**) and AC1610F1 (**11**), and to a lower extent Sib (**1**), exhibited a robust ability to inhibit CuOOH-induced cell death both in nasal epithelial cells from healthy donors and cystic fibrosis patients (Fig. 6A–C).

### Dual effect of sibiriline on the neurotoxicity induced by 6-OHDA

**Fig. 7:**
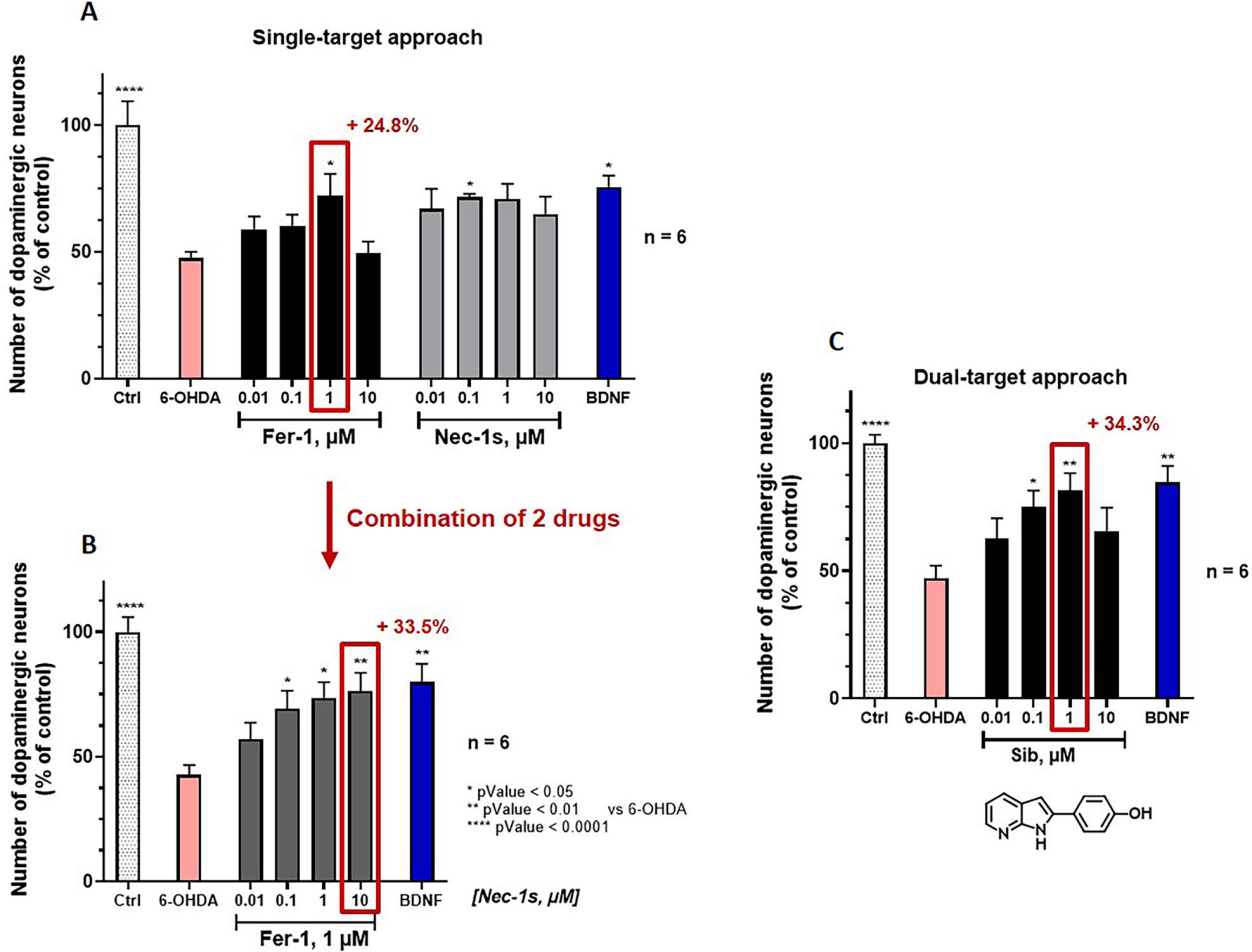
Sib (1) protects neurons in a Parkinson model of 6-OHDA-induced rat dopaminergic neurons degeneration. Primary rat dopaminergic neurons were exposed to 20 µM of 6-OHDA and several doses of Fer-1 or Nec-1s (**A**) or Sib (**C**) or a combination of Fer-1 + Nec-1s (**B**) for 48 h. Cells were fixed and labeled with antibody against tyrosine hydroxylase (TH). Results were expressed as a percentage of the number of TH positive dopaminergic neurons compared to the control non-treated condition. Data were expressed as mean ± SEM of six replicates. A global analysis of the data was performed using one-way ANOVA following by Dunnett’s test. The level of significance is set at *p* < 0.05.

To illustrate the superior neuroprotective effect of a dual inhibitor compared to the simultaneous use of two separate cell death inhibitors, we next used a well-established cell-based model of Parkinson’s disease: primary rat dopaminergic neurons exposed to 6-hydroxydopamine (6-OHDA) for 48 hours. This model has previously been shown to involve either necroptosis [37] or ferroptosis [38] pathways, but not both simultaneously. Treatment with 20 µM 6-OHDA for 2 days induced a marked loss of dopaminergic neurons, while the reference neurotrophic factor BDNF (50 ng/mL) significantly attenuated this loss. Figure 7A, B presents the effects of Nec-1s and Fer-1 used individually or in combination in concentrations ranging from 0.01–10 µM. Fer-1 alone exhibited a significant neuroprotective effect at 1 µM, while other concentrations showed no significant effect (Fig. 7A). Nec-1s alone provided partial protection only at 0.1 µM (Fig. 7A). In combination, the greatest protective effects were achieved with 1 µM Fer-1 and increasing concentrations of Nec-1s (Fig. 7B); both showed dose-dependence of their effects. Significant dose-dependent protection was observed with 0.1–10 µM of Nec-1s combined with 1 µM of Fer-1, with maximal efficacy at 10 µM Nec-1s; this treatment resulted in a 33.5% increase in dopaminergic neuron survival compared to the 6-OHDA condition. Figure 7C shows that Sib (**1**), provided robust and dose-dependent neuroprotection with maximal protection at 1 µM, leading to a 34.5% increase in dopaminergic neurons. These results demonstrate that sibiriline, a dual necroptosis and ferroptosis inhibitor, offers effective neuroprotection in this Parkinson’s disease model and suggest that one must focus on concurrent inhibition of both necroptosis and ferroptosis pathways in order to achieve optimal neuronal survival. This conclusion is corroborated by the apparently synergistic effects observed with the combination of Fer-1 and Nec-1s.

## Discussion and conclusion

Sibiriline (**1**) was previously recognized as an inhibitor of the necroptotic regulated cell death pathway. In the present study, we confirmed that Sib (**1**) is a potent RIPK1 inhibitor and we showed that among the kinases putatively targeted by Sib (**1**) (see [8] for details), RIPK1 is the most affected in the cell with millimolar concentrations of ATP. In addition, we characterized Sib as a novel ferroptotic cell death inhibitor. In line with this new activity, we showed by transcriptomic assays that HMOX1, PTGS2, SLC7A11, MT1G and GCLC genes, all of which were already known to be involved in ferroptosis pathways, are downregulated by treatment with Sib (**1**). Metallothionein-1G (MT1G) is a ferroptosis-related gene involved in the regulation of oxidation and metalloproteinases. Interestingly, the MT1G gene was also reported as a sensitive dynamic biomarker of neurotoxicity (induced by neurotoxicants including 6-hydroxydopamine or paraquat) in LUHMES immortalized neurons [39]. The change in expression of PTGS2, which encodes cyclooxygenase 2 (COX2), can be observed in both ferroptotic or non-ferroptotic conditions or during inflammatory conditions [40]. NFE2-like bZIP transcription factor 2 (NRF2), a master transcription factor of the antioxidant response, is known to regulate the expression of genes involved in the control of ferroptosis, including HMOX1, SLC7A11 and GCLC, with SLC7A11 (encodes for xCT cystine-glutamate antiporter) and GCLC (encodes for glutamate–cysteine ligase catalytic subunit) directly linked to the biogenesis of glutathione (GSH). Note here that HMOX1, the biomarker of ferroptosis inhibition most affected by Sib treatment, is known to be induced in response to oxidative stress [40–42]. The decrease of HMOX1 expression by Sib treatment highlights its protective effect on the cells. While none of these genes can be considered as specific ferroptosis biomarkers, our work suggests that together they may define a ferroptotic signature.

We next analyzed the mechanism of action of Sib (**1**) using the FENIX assay for the assessment of RTA activity in phospholipid bilayers. We showed that Sib (**1**) is an RTA and that the efficacy of the molecule derives primarily from the phenolic moiety. As reported in Shah et al. [32], the quantitative data derived from the FENIX assay, namely the apparent inhibition rate constant (*k*_inh_), is generally predictive of the anti-ferroptotic potency of RTAs. In this study, we also observe a correlation between the apparent rate constant of inhibition derived using FENIX and potency at subverting ferroptotic cell death. Indeed, the most active RTA detected using the FENIX assay, compound (**11**), was the most cytoprotective against RSL3-induced ferroptosis in a multitude of cell lines (see Table 1 and S1). The primary anti-ferroptotic function of this compound is therefore presumably to slow lipid peroxidation by trapping (phospho)lipid-derived peroxyl radicals. Although the most potent ferroptosis inhibitors have RTA activity, we want to underline that compounds, such as enzymatic modulators (non-RTA LOX inhibitors, ACSL4 inhibitors or GPX4 activators), act through alternative mechanisms [43].

Taken together, our results indicate that sibiriline (**1**) is a new chemical agent acting on both necroptosis and ferroptosis RCD pathways, and that it can be used in polypharmacological-based therapies. Sib (**1**) is a new member of the class of dual RIPK1-RTA inhibitors, a class that we propose to call “**RIPROStatins**”; that is, dual Receptor Interacting Protein Kinase inhibitors also acting as (phospho)lipid ROS trapping agents. Therefore, the well-known RIPK1 inhibitor, Nec-1, which was previously shown to has RTA activity [44] is by definition a member of the RIPROStatins class of compounds. The SAR study presented here shows that it is possible to design highly active RTA compounds (such as **11**) that may also pave the way for the management of complex diseases whose medical needs are currently unmet, such as chronic neurodegenerative pathologies.

## Materials and methods

### Chemistry

Nuclear magnetic resonance (NMR) spectra were recorded on a Bruker AV300 NMR spectrometer using TMS as an internal standard and chemical shift values (δ) are expressed in ppm relative to deuterated solvent as the internal standard. High-resolution mass spectra (HRMS) were recorded on a hybrid quadrupole time-of-flight (QTOF) mass spectrometer (Impact II, Bruker) equipped with an electrospray ionization ion source (ESI) in positive (or negative) ion mode. All reactions were monitored by thin-layer-chromatography (TLC) on silica gel plates. Chromatographic purification was conducted on commercial silica gel (60, particle size 40-63 μm). All reagents and dry solvents were purchased from commercial suppliers and used without further purification unless otherwise noted. Glassware used for all moisture/oxygen sensitive manipulations was dried at 100°C in an oven, evacuated on a Schlenk line and placed under an argon atmosphere. The synthesis of Sib (**1**) and Sib-f (**6**) were already described in Le Cann et al. [8] for Sib; and Bali et al. [45] for Sib-f. The general procedures for synthesis of sibiriline-Me, sibiriline-f-Me, AC1535, AC1584 and AC1610F1 are described in Table S2. ^1^H NMR, ^13^C NMR spectra and HRMS analysis for these compounds are reported as supplementary Figs. S7–S21.

### Inhibited co-autoxidations of egg PC liposomes (FENIX)

Bulk lipid mixture (290 µL) was added to the wells of a Nunc black polypropylene round-bottomed 96-well microplate. Bulk lipid mixture contained egg PC liposomes (1.03 mM), prepared as in [44] and STY-BODIPY (1.03 µM) in chelex-treated phosphate buffered saline (cPBS) (12 mM phosphate, 150 mM NaCl, pH 7.4). Using a 1-10 µL multi-channel pipette, 5 µL of inhibitor stock solution (in DMSO) or DMSO vehicle was added to wells and manually mixed with a 100-300 µL multichannel pipette set to 250 µL. The plate was then inserted into a BioTek H1 Synergy microplate reader equilibrated to 37 °C and incubated for 10 min. Following incubation, 5 µL of DTUN solution in ethanol (12 mM) was added to each well using a 1-10 µL multi-channel pipette to afford a final volume of 300 µL (final concentrations: 1 mM egg PC liposomes, 1 µM STY-BODIPY, 0.2 mM DTUN, concentration of inhibitor varies between 4 and 32 µM). The reaction mixtures were manually mixed again with a 100-300 µL multichannel pipette set to 250 µL and re-inserted into the microplate reader. The plate was vigorously shaken (double orbital shake) for 1 minute followed by a 3.5 min delay. Fluorescence was then recorded (λ_ex_ = 488 nm; λ_em_ = 518 nm; gain = 60) every minute for 6 hours. Data was transformed and kinetic parameters were derived as in [32].

### Biological evaluations

#### Reagents

Ferrostatin-1 (Fer-1/F1), erastin, Ras-selective lethal 3 (RSL3) were obtained from Selleck Chemicals (Houston, TX, USA). Necrostatin-1s was from Calbiochem (San Diego, CA, USA), mouse TNF-α was from Bio-Techne (Minneapolis, MN, USA), z-VAD.fmk was from MedChemExpress (Monmouth Junction, NJ, USA), H2DCFDA and BODIPY 581/591 C11 dyes were from Invitrogen (Carlsbad, CA, USA). FIN56, and DMSO (Dimethylsulfoxide) were from Merck Life Science (Darmstadt, Germany). FINO_2_ was purchased from Bertin Bioreagent (Montigny le Bretonneux, France).

### Cell culture

Human neuroblastoma SH-SY5Y cell line, human fibrosarcoma HT1080 cell line, mouse fibroblast NIH3T3 cell line and mouse hippocampal HT-22 cell line were originally obtained from American Type Culture Collection (ATCC, Manassas, VA, USA). Pfa1 cells described in Seiler et al. [46] and NIH3T3 described in Tonnus et al. [26] were shared by the laboratory of Dr. Marcus Conrad and Pr. Andreas Linkermann, respectively. HT1080 cells were maintained in Dulbecco’s Modified Eagle’s Medium F12 (DMEM F12) with 10% Foetal Bovine Serum (FBS) and all the others cell lines were maintained in DMEM supplemented with 10% FBS. Cells were cultured at 37 °C under a humidified 5% CO_2_ atmosphere. Medium and serum were purchased from Thermo Fisher Scientific (Gibco, Waltham, MA, USA).

### RNAseq transcriptomic study

SH-SY5Y cells were seeded in 6-well plates at 1.2 ×10^6^ cells per well. After overnight incubation, cells were treated for 4 hours with 5 µM RSL3, 20 µM Sib or vehicle, alone or in combination, with three replicates for each condition. After treatment RNA was extracted using RNeasy plus mini kit and QIAshredder (QIAGEN GmbH, Hilden, Germany) following the manufacturer’s instructions. RNA quantification was performed with a nanodrop spectrophotometer and quality control was carried out using a Bioanalyser. The libraries were prepared following the Stranded mRNA Prep protocol from Illumina, starting with 600 ng of high-quality total RNA. RNA-seq libraries were sequenced (2 × 59 bp, Illumina NovaSeq 6000), aligned to the Ensembl release 101 reference genome using STAR (v2.7.6a), and quantified with RSEM (v1.3.1). Differential expression analysis was performed using DESeq2 (v1.26.0) with standard normalization (median-of-ratios) and Wald test, following Love et al. [47]. Genes with ≥10 reads in ≥3 samples were retained. R scripts and parameters are available online on the platform, https://github.com/GENOM-IC-Cochin/RNA-Seq_analysis.

### Phenotypic analysis of necroptosis and ferroptosis cell death

Cell-based necroptosis assay was performed using the mouse fibroblast NIH3T3 cell line. Necroptosis cell death was induced with a combination of 5 ng/ml of mouse tumor necrosis factor TNF-α and 20 µM of z-VAD.fmk.

Cell-based ferroptosis assays were performed using the human neuroblastoma cell line SH-SY5Y and the mouse NIH3T3 cell line. Cells were seeded in 96-well plates at a density of 10^5^ cells/mL and ferroptosis was induced with different classes of ferroptosis inducers (FINs); namely erastin, RSL3, FIN56 or FINO_2_ (representing class I to IV, respectively). Each inhibitor or the vehicle control was added concomitantly with cell death inducers. Ferrostatin-1 (Fer-1/F1) at a concentration of 1 µM was used as a positive control. Cell viability and cytotoxicity were assessed after 16-24 hours of treatment, depending of the cell line using the MTS assay (CellTiter 96^®^ AQueous Non-Radioactive Cell Proliferation Assay; Promega, Fitchburg, WI, USA) or the LDH (lactate dehydrogenase) Cytotoxicity assay kit (Invitrogen, Carlsbad, CA, USA), respectively, and following manufacturer recommendations. Unless otherwise indicated, experiments were carried out in triplicate and repeated at least two times.

### Measurement of total intracellular ROS levels

H2DCFDA (2′,7′-dichlorofluorescin diacetate) was used as a cell-permeable probe for intracellular ROS. SH-SY5Y cells were seeded in complete medium in a 12-well plate at 4 × 10^5^ cells per well, at 37 °C and 5% CO_2_. After 24 hours, cells were treated with Sib (10 µM), RSL3 (5 µM), or Sib+RSL3 for 5 hours. Cells were incubated with 5 µM of H2DCFDA for 30 min at 37 °C and protected from light. Cells were trypsinized, centrifuged and resuspended in PBS. ROS detection, i.e. H2DCFDA positive cells, was performed using an Attune NxT flow cytometer (Thermo Fisher Scientific, Waltham, MA, USA) using the 488 nm laser for excitation and monitored at 535 nm. For each analysis, 10,000 events were recorded.

### Measurement of lipid peroxidation

SH-SY5Y cells were seeded in 96-well black plates with a clear bottom (Corning, Big Flats, NY, USA) at a density of 10,000 cells per well. Ferroptosis was induced with 5 µM RSL3. The lipid peroxidation assay was performed as described by Delehouze et al. [25]. The IncuCyte S3 live-cell imaging and analysis system (Essen BioScience, Sartorius, Göttingen, Germany) was used to capture images using a 10X objective and 440–480 nm Excitation/504–544 nm Emission filters at hourly intervals over the course of 24 hours. For each condition, the fluorescence of nine replicates was recorded with the cell imaging apparatus.

### Cystic fibrosis cell model

Patients’ primary Human Nasal Epithelial Cells (HNECs) were collected on superior turbinates using smear brushes (Hospital of Toulouse, France) as previously described [35]. Cells were collected in medium and then centrifuged for 5 min 400 g at 4 °C. Cells were resuspended in 4 mL TrypLE Express (GIBCO) + 20 µL Sputolysin (200X) and incubated at 37 °C for 5 min to disrupt mucus. TrypLE was diluted with 4 mL of Advanced DMEM F-12.

Cells were expanded in medium Pneumacult. After a week of proliferation, basal cells were counted and seeded onto collagen-coated (0.03 mg/mL) and maintained in Pneumacult Ex Plus Medium (StemCell) at 37 °C 5% CO_2_. pHNECs were seeded one day before the stimulation in 96-well plates at 30,000 cells per well in 200 µL of Pneumacult medium. Cell’s medium was changed to OPTIMEM the day of the experiment, and cells were treated or not with the indicated inhibitors (described in legend of Fig. 6) for 1 h before the stimulation with Cumene Hydroperoxide (CuOOH, 50 µM, 8 h). Cell lysis was measured by determining the LDH release into the cell supernatant with the LDH CyQUANT kit (Thermo Fisher Scientific). 50 µL cell supernatant was co-incubated with 50 µL LDH substrate for 15-30 min at room temperature in dark. The enzymatic reaction was ended with 50 µL of stop solution. Maximal cell death was quantified with whole cell lysates from unstimulated cells exposed to 1% Triton X-100. Experiments were performed in triplicate and repeated at least three times.

### 6-OHDA Parkinson’s model

Rat dopaminergic neurons were cultured as described by Lavigne et al. [48]. 6-Hydroxydopamine hydrobromide (6-OHDA) was purchased commercially (Tocris, Ref: 2547/50) and dissolved in culture medium at a concentration of 40 µM. After 7 days of culture, rat primary mesencephalic neurons were pre-treated for 1 hour with Sib, Nec-1s or Fer-1 (10 µM, 1 µM, 0.1 µM and 0.01 µM) alone or combination of Nec-1s and Fer-1 at all concentrations and reference compound (BDNF at 50 ng/mL) and then exposed to 6-OHDA at a final concentration of 20 µM for 2 days in order to induce a neuronal cell death of about 50% +/-5%. After 2 days of intoxication, cells were proceeded for the detection of Tyrosine Hydroxylase (TH) by immunofluorescence as described by Lavigne et al. [48]. Briefly, cells were incubated with a mouse monoclonal anti-Tyrosine Hydroxylase antibody (TH, 1/10 000, Sigma; ref: T1299) in a solution of PBS overnight at 4 °C. Staining was revealed with the addition of an Alexa Fluor 488 goat anti-mouse IgG (1/400, Molecular probe, ref: A11001) in PBS with 1% FCS and 0.1% saponin for 1 hour at room temperature. Nuclei of cells were labeled by a fluorescent marker (Hoechst, Sigma; ref: B1155). For each analysis, images were taken automatically with automated microscope InCell2200 (GE healthcare) with the objective 20x. The same corresponding area was acquired in each well avoiding any experimenter impact on the acquisition process. Analyses of the number of dopaminergic neurons (TH positive neurons) and the neurites length of dopaminergic neurons (TH positive neurites), were then done automatically using the same program for all treatment conditions so the same selected parameters are applied with Image Analysis Software (Toolbox processing Developer v1-9-2, GE healthcare). The sum of each measured parameter was done on 20 images per well. The value obtained for each condition was then normalized to control conditions. Data were expressed as the mean ± standard error of the mean (N = 6 per condition) and analyzed using one-way ANOVA followed by Dunnett correction.

### Protein kinase inhibition assays

The enzymatic activity of ABL1 was measured using the ADP-Glo^TM^ bioluminescent kinase assay kit (Promega, Madison, WI) according to the recommendations of the manufacturer and as described in [49]. RIPK1, AURKC, DRAK2, JAK2, KIT and PDGFR-β kinases were analyzed by the Eurofins company (Celle L’Evescault, France), following protocols reported on their website.

### Statistical analyses

Data from a minimum of two experiments were expressed as means ± SEM. Statistical analyses were done by ANOVA, Tukey’s Multiple Comparison Test and Student’s *t* test for two groups of data, and significance levels used are *P < 0.05, **P < 0.01, ***P < 0.001, ****P < 0.0001 by using GraphPad Prism 10 software (GraphPad Software, San Diego, CA, USA).

## Supplementary information

Supplementary Information

## Data Availability

Data will be made available on request.
