# Supplementary material for: Sibiriline, a novel dual inhibitor of necroptosis and ferroptosis, prevents RIPK1 kinase activity and (phospho)lipid peroxidation as a potential therapeutic strategy
Source: Cell Death Discov. 2025 Nov 28;11:552. doi: 10.1038/s41420-025-02852-8 (PMC12663298; doi:10.1038/s41420-025-02852-8)
Supplement: Supplementary file 1 — Supplementary Information [file 41420_2025_2852_MOESM1_ESM.docx]

**SUPPLEMENTARY INFORMATION**

**Sibiriline, a novel dual inhibitor of necroptosis and ferroptosis, prevents RIPK1 kinase activity and (phospho)lipid peroxidation as a potential therapeutic strategy**

Claire Delehouzé ^1,2^, Melodie Mallais ^3^, Arnaud Comte ^4^, Romain Lucas ^1^, Blandine Baratte ^2,5^, Sophie Bélal ^1^, Axelle Autret ^1^, Nathalie Py ^6^, Rémy Steinschneider ^6^, Lucie Adoux ^7^, Benjamin Saintpierre ^7^, Franck Letourneur ^7^, Thomas Robert ^2,5^, Céline Cougoule ^8^, Caio Bomfim ^8^, Rémi Planès ^8^, David Péricat ^8^, Jeannette Chloë Bulinski ^2^, Marie-Thérèse Dimanche-Boitrel ^9^, Peter Goekjian ^10^, Etienne Meunier ^8^, Morgane Rousselot ^1^, Derek A. Pratt ^3^, Stéphane Bach ^2,5,^*

^1^ SeaBeLife Biotech, Place Georges Teissier, 29680, Roscoff, France

^2^ Sorbonne Université, CNRS, Laboratoire de Biologie Intégrative des Modèles Marins, LBI2M, F-29680 Roscoff, France

^3^ Department of Chemistry and Biomolecular Sciences, University of Ottawa, Ottawa, Ontario, Canada

^4^ Université de Lyon, CNRS UMR 5246, ICBMS, Chimiothèque, Université Claude Bernard Lyon 1, F-69622 Villeurbanne, France

^5^ Sorbonne Université, CNRS, Kinase Inhibitor Specialized Screening facility, KISSf, F-29680 Roscoff, France

^6^ Neuron Experts, Cité de la cosmétique, 2 rue Odette Jasse, 13015, Marseille, France

^7^ Plateforme GenomIC, Université de Paris, Institut Cochin, INSERM-CNRS, F-75014, Paris, France

^8^ Institut de Pharmacologie et Biologie Structurale (IPBS), Université de Toulouse, CNRS, Toulouse, France

^9^ France Univ Rennes, Inserm, EHESP, Irset (Institut de Recherche en santé, environnement et travail) - UMR_S 1085, F-35000 Rennes, France

^10^ Université de Lyon, CNRS UMR 5246, ICBMS, Laboratoire Chimie Organique 2-Glycosciences, Université Claude Bernard Lyon 1, F-69622 Villeurbanne, France

* **Corresponding author:**

Stéphane Bach, CNRS, Sorbonne Université, UMR8227 - LBI2M, Station Biologique, Place Georges Teissier, F-29688 Roscoff, France. Tel: +33 2 98 29 23 91; Fax: +33 2 98 29 25 26; Email: bach@sb-roscoff.fr

**
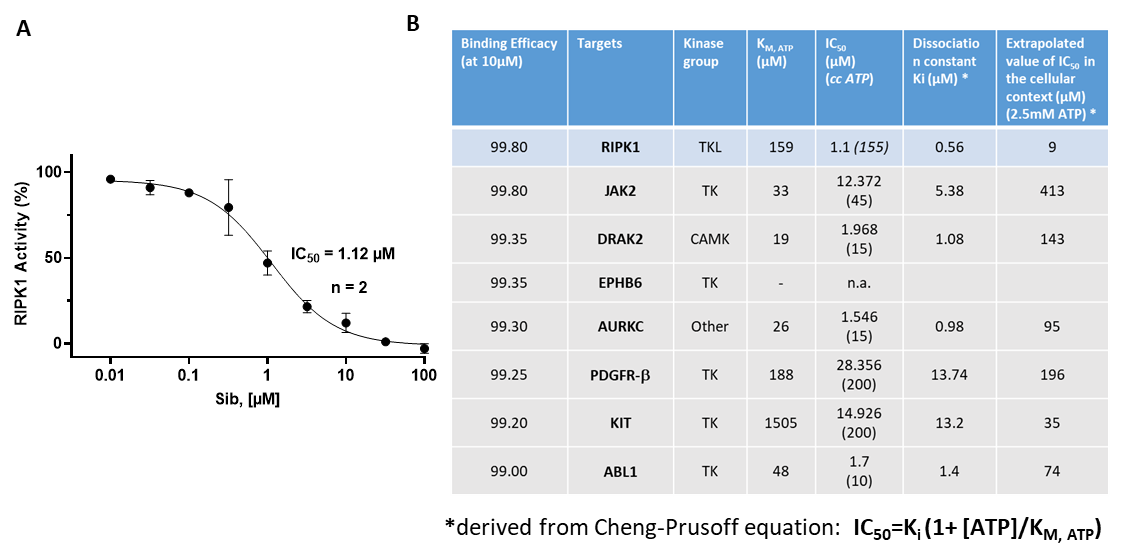
**

**Fig. S1 Selectivity analysis against a panel of kinases of sibiriline (Sib, 1), a RIPK1 inhibitor.** (**A**) Analysis of the dose-dependent effect of Sib on the kinase activity of human RIPK1. The enzyme was tested with 155 µM of ATP, corresponding to the K_M_, _ATP_ value of RIPK1. (**B**) Analysis of the selectivity of sibiriline against a selected panel of kinases that were found to interact with sibiriline (see Le Cann et al. 2017, doi: 10.1111/febs.14176., for experimental details). The binding efficacy values reported in the first column represent the percentage efficacy of competition with 10 µM of sibiriline: 100 % would indicate that all of the target was competed with the tested compound, showing a strong binding efficacy.

**
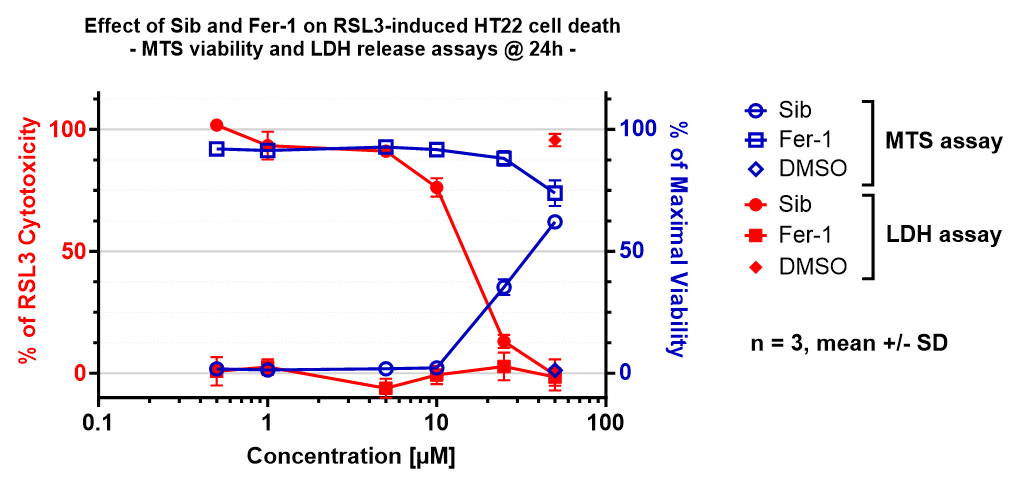
**

**Fig. S2 RSL3-induced ferroptosis assay on mouse hippocampal cell line HT22.** HT22 neuronal cells were seeded in 96-well plate at a density of 5000 cells/well before being co-treated 24 h with 1 µM of RSL3 and increasing concentrations of Sib (**1**) or ferrostatin-1 (Fer-1). Cell death was estimated by lactate dehydrogenase (LDH) release assay. Results are plotted in % of LDH release measured when cells are treated with RSL3 (left axis, colored red). Cell viability was evaluated by MTS reduction assay. Results obtained (colored blue) were plotted in % of maximal viability with DMSO-treated cells (right axis). Data are shown as the mean +/- SD of three replicates.

**Fig. S3 Identification of sibiriline indole derivative, Sib-f (6), as potent ferroptosis inhibitor.** Dose-dependent inhibition of RSL-3-induced ferroptosis by increasing concentrations of Sib-f (6). After a 24-h incubation, the effect of the tested compound on the cell viability of SH-SY5Y neuroblastoma cells was evaluated by MTS (3-(4,5-dimethylthiazol-2-yl)-5-(3-carboxymethoxyphenyl)-2-(4-sulfophenyl)-2H-tetrazolium) reduction assay and is expressed in % of maximal viability detected for cells treated with DMSO (n= 2, mean +/-SEM). The chemical structure of Sib-f is depicted on the figure.

**
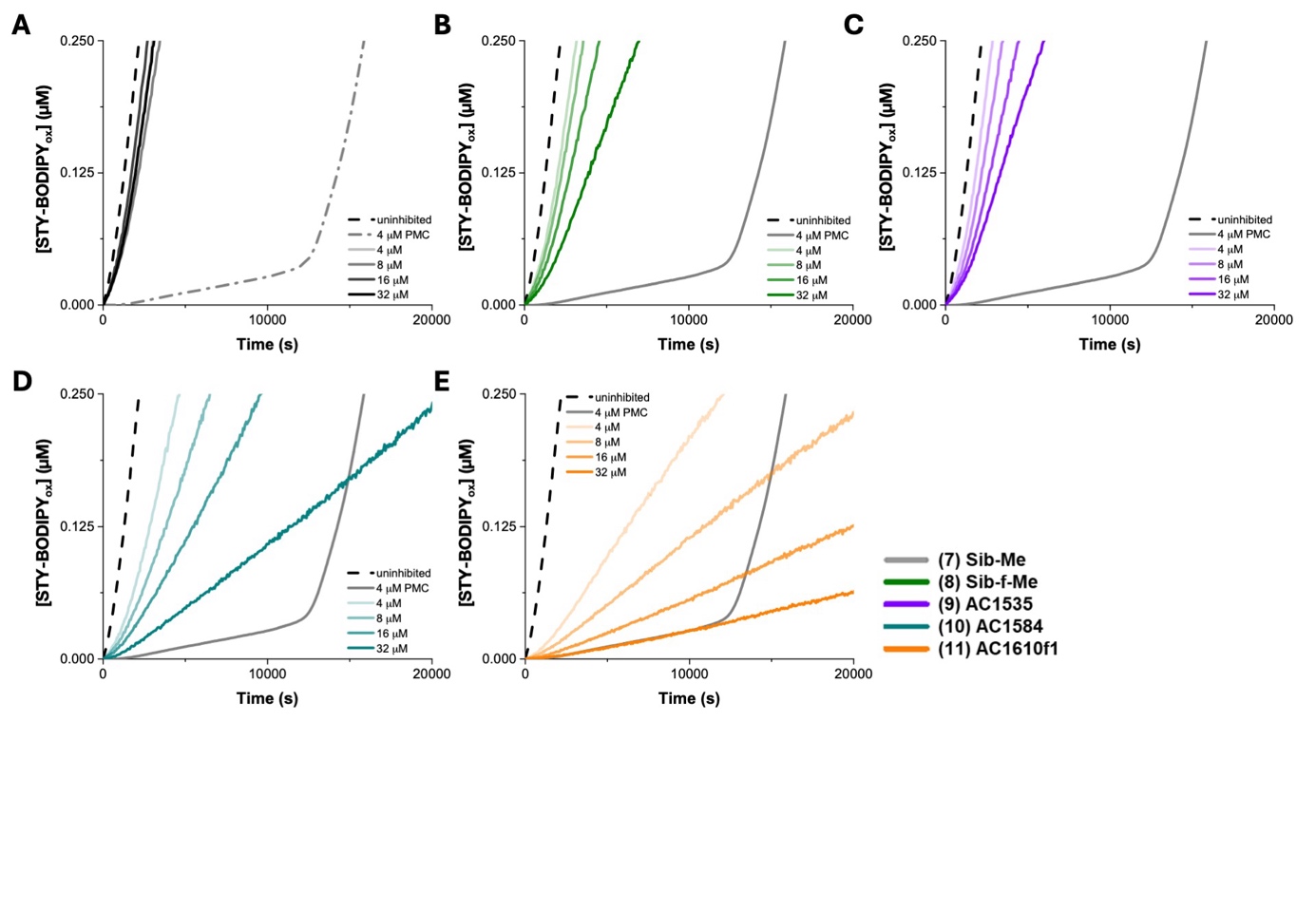
**

**Fig. S4 Evaluation of (phospho)lipid peroxidation-inhibiting activity of sibiriline (1) derivatives by the FENIX assay.** Phosphatidylcholine liposome (1 mM) co-autoxidations in the presence of STY-BODIPY (1 µM) and initiated with DTUN (0.2 mM) inhibited by 4-32 µM (**A)** Sibiriline-Me (**7**), (**B)** Sibiriline-f-Me (**8**), (**C)** AC1535 (**9**), (**D**) AC1584 (**10**), or (**E**) AC1610F1 (**11**). Uninhibited PC liposome co-autoxidations represented in black (dashed) and 4 µM PMC inhibited co-autoxidations represented in grey.

**
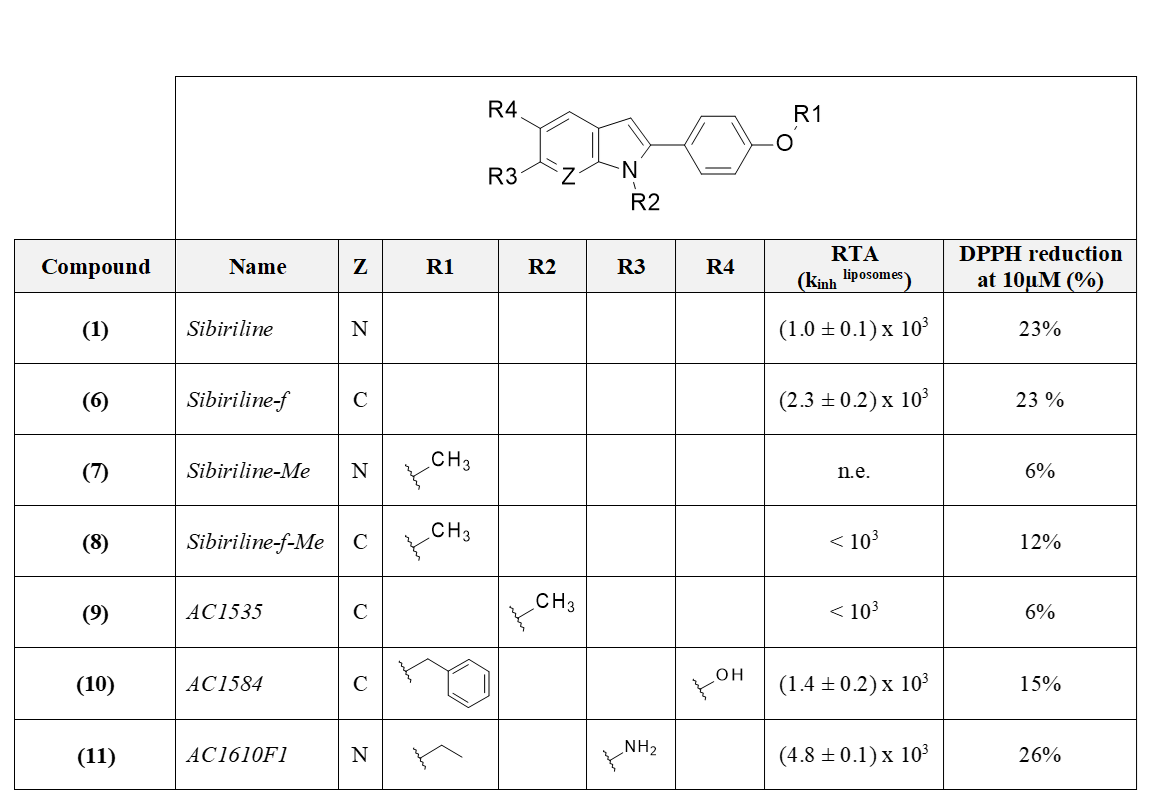
**

**Table S1** **Measure of antioxidant activity of sibiriline (1) and its derivatives using the DPPH (2,2-Diphenyl- 1-picrylhydrazyl) reduction assay.** DPPH was prepared at 15.85 µM in 90% methanol and added to several concentrations of compounds in 96-well plate (200µL per well). The reaction was conducted at room temperature for 30 min and absorbance was measured at 517 nm using the EnVision microplate reader (PerkinElmer, Waltham, MA, USA). Tocopherol, a well-known antioxidant, was used as control. The percentage of DPPH reduction was calculated by dividing the difference between the absorbance of DPPH and those of compounds by the absorbance of DPPH, and multiplying by 100. Three independent biological replicates were performed for the experiment. n.e.: no significant effect detected up to 32 µM

**
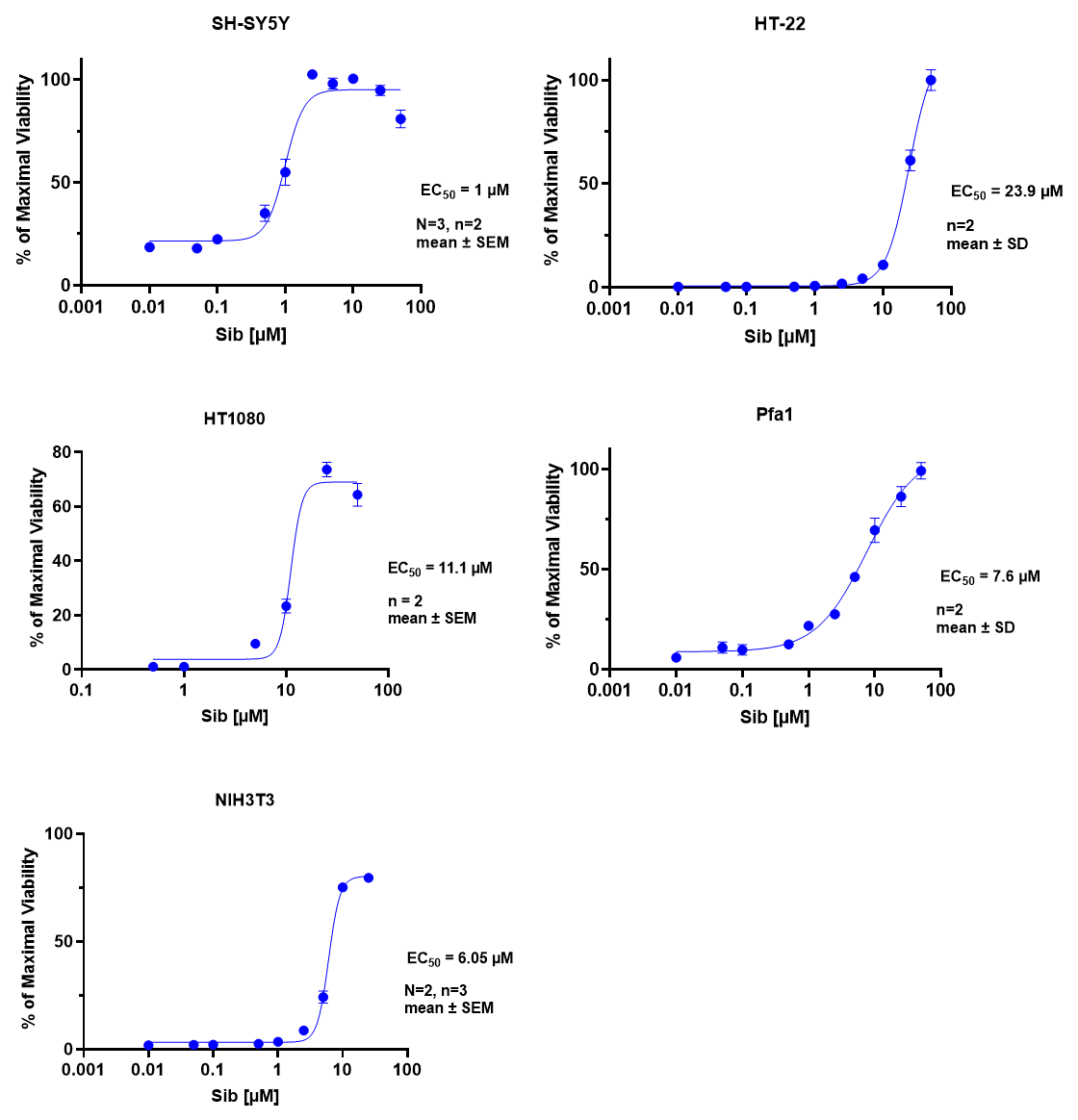
**

**Fig. S5 Sibiriline (1) protects SH-SY5Y, HT22, HT1080, Pfa1 and NIH3T3 cell lines from ferroptotic cell death triggered by RSL3.** Cells were co-treated 24 h with RSL3 (at 5µM for SHSY-5Y and 1µM for the other cell lines) and increasing concentrations of sib. 100% of viability was determined by treating the cells with a similar dose of DMSO without sibiriline and RSL3. EC_50_ were determined from the dose-response curves using GraphPad PRISM software . Cell viability was estimated by MTS assay. Data are shown as the mean ± SEM of six replicates.

**
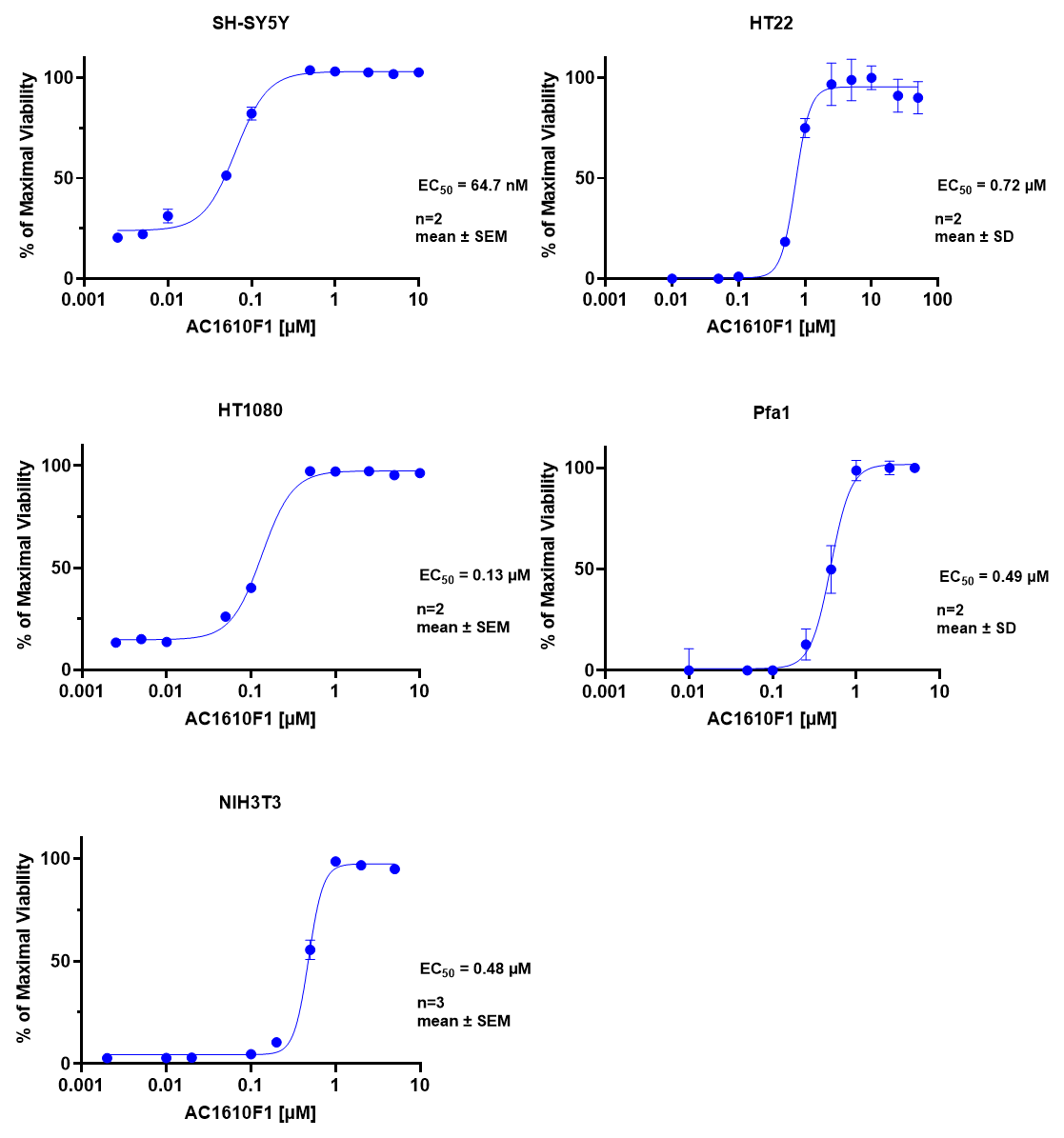
**

**Fig. S6 AC1610F1 (11) protects SH-SY5Y, HT22, HT1080, Pfa1 and NIH3T3 cell lines from ferroptotic cell death triggered by RSL3.** Cells were co-treated 24 h with RSL3 (at 5µM for SHSY-5Y and 1µM for the other cell lines) and increasing concentrations of AC1610F1 (**11**). 100% of viability was determined by treating the cells with a similar dose of DMSO without sibiriline and RSL3. EC_50_ were determined from the dose-response curves using GraphPad PRISM software . Cell viability was estimated by MTS assay. Data are shown as the mean ± SEM of six replicates.

| **Compound** | **General procedure for synthesis** | **^1^H NMR** | **^13^C NMR** | **HRMS** |
| --- | --- | --- | --- | --- |
| **Sibiriline-Me (7)** | LDA (1.95 ml of 2.0 M solution, 3.9 mmol, 1.8 eq) was dissolved in anhydrous THF (10 ml) at -5°C under argon and a solution of 3-picoline (200 mg, 2.2 mmol, 1 eq) in anhydrous THF (10 ml) was added dropwise at 0°C. The orange mixture was stirred for 20 min before dropwise addition of a solution of 4-methoxybenzonitrile (316 mg, 2.2 mmol, 1 eq) in anhydrous THF (10 ml). After 1h at 0°C, more LDA (1.95 ml of 2.0 M solution, 3.9 mmol, 1.8 eq) was added dropwise and the reaction was slowly warmed to RT during 1h before being heated to reflux for 2h. After cooling to RT, the yellow solution was quenched carefully with saturated NH_4_Cl (10 ml) and water (40 ml) was added. The precipitate was filtered, washed with diethyl ether, water and dried under vacuum to afford a light yellow solid (327 mg, 68%). | ^1^H NMR (300 MHz, DMSO-d_6_, **Fig. S7**) δ 12.03 (s, 1H), 8.16 (dd, *J* = 4.7, 1.6 Hz, 1H), 7.99 – 7.77 (m, 3H), 7.05 – 7.01 (m, 3H), 6.77 (d, *J* = 2.0 Hz, 1H), 3.80 (s, 3H). | ^13^C NMR (75 MHz, DMSO-d_6_, **Fig. S8**) δ 159.2, 149.6, 142.2, 138.4, 127.3, 126.8, 124.2, 121.2, 115.9, 114.4, 95.7, 55.2. | HRMS (ESI, **Fig. S9**): m/z calculated for C_14_H_12_N_2_O [M+H]^+^ 225.1022, found 225.1025. |
| **Sibiriline-f-Me (8)** | Phenylhydrazine (1.8 ml, 18.5 mmol, 1 eq) and 4-methoxyacetophenone (2.78 g, 18.5 mmol, 1 eq) were dissolved in ethanol (40 ml) and 5 drops of glacial acetic acid were added. The mixture was heated to reflux for 2h, cooled to RT and concentrated under vacuum to obtain the intermediate imine as a yellow solid. This crude product was suspended in Eaton’s reagent (approx. 30 ml) and the mixture was heated to 90°C for 1h30. The resulting black solution was cooled to RT, added to ice water (200 ml) and neutralized by slow addition of concentrated NaOH. The precipitate was filtered, washed with water and dried under vacuum to obtain a brown solid. Purification was performed by recrystallization from toluene to afford the desired compound as a light brown solid (2.3 g, 56%). | ^1^H NMR (300 MHz, DMSO-d_6_, **Fig. S10**): δ 11.41 (s, 1H), 7.79 (d, *J* = 6.8, 2H), 7.49 (d, *J* = 7.7 Hz, 1H), 7.37 (d, *J* = 7.7 Hz, 1H), 7.13 – 6.91 (m, 4H), 6.76 (d, *J* = 2.1 Hz, 1H), 3.81 (s, 3H). | ^13^C NMR (75 MHz, DMSO-d_6_, **Fig. S11**) δ 158.8, 137.8, 136.9, 128.8, 126.4, 124.90, 121.1, 119.7, 119.3, 114.4, 111.1, 97.3, 55.2. | HRMS (ESI, **Fig. S12**): m/z calculated for C_15_H_13_NO [M+H]^+^ 224.1070, found 224.1071. |
| **AC1535 (9)** | N-methylindole (190 μL, 1.52 mmol, 1 eq) was placed in a 25 ml flask under argon, along with Pd(OAc)_2_ (17 mg, 0.08 mmol, 0.05 eq), Ag_2_O (283 mg, 1.22 mmol, 0.8 eq), 4-iodophenol (669 mg, 3.04 mmol, 2 eq) and 2-nitrobenzoic acid (381 mg, 2.28 mmol, 1.5 eq). Dry DMF (8 ml) was added and the reaction was stirred at RT for 16h. The mixture was filtered on Celite^®^, the cake was washed with EtOAc and brine was added. The aqueous phase was extracted with EtOAc, the organic extracts were washed with brine, dried over MgSO_4_ and concentrated. Purification by flash chromatography (95:5 to 80:20 PE:EtOAc) afforded a beige solid (139 mg, 36%). | ^1^H NMR (300 MHz, CDCl_3_, **Fig. S13**) δ 7.62 (d, *J* = 7.7 Hz, 1H), 7.38 (d, *J* = 8.6 Hz, 2H), 7.35 (d, *J* = 7.7 Hz, 1H), 7.26 – 7.20 (m, 1H), 7.16 – 7.10 (m, 1H), 6.93 (d, *J* = 8.6 Hz, 2H), 6.50 (d, *J* = 0.8 Hz, 1H), 3.72 (s, 3H). | ^13^C NMR (75 MHz, CDCl_3_, **Fig. S14**) δ 155.5, 141.5, 138.3, 131.0, 128.1, 125.60, 121.6, 120.4, 120.0, 115.6, 109.7, 101.1, 31.18. | HRMS (ESI, **Fig. S15**): m/z calculated for C_15_H_13_NO [M+H]^+^ 224.1070, found 224.1074. |
| **AC1584 (10)** | 5-hydroxyindole (311 mg, 2.34 mmol, 1 eq) and 4-benzyloxyphenyl boronic acid (800 mg, 3.5 mmol, 1.5 eq) were placed in a 25 ml flask and dissolved in acetic acid (10 ml). Pd(OAc)_2_ (52 mg, 0.23 mmol, 0.1 eq) was added and the mixture was vigorously stirred at RT under air for 20h. The reaction was quenched by careful addition to saturated NaHCO_3_ and extracted with a mixture of EtOAc:iPrOH (8:2). The organic extracts were washed with brine, dried over MgSO_4_ and concentrated to a black solid. Purification by flash chromatography (100:0 to 80:20 CH_2_Cl_2_:EtOAc) afforded a light brown powder that was triturated in CH_2_Cl_2_ (5 ml) and filtered to obtain an off-white solid (115 mg, 16%). | ^1^H NMR (300 MHz, DMSO-d_6_, **Fig. S16**) δ 11.07 (d, *J* = 2.2 Hz, 1H), 8.61 (s, 1H), 7.73 (d, *J* = 8.7 Hz, 2H), 7.50 – 7.33 (m, 5H), 7.15 (d, *J* = 8.6 Hz, 1H), 7.10 (d, *J* = 8.7 Hz, 2H), 6.80 (d, *J* = 2.3 Hz, 1H), 6.60 – 6.55 (m, 2H), 5.15 (s, 2H). | ^13^C NMR (75 MHz, DMSO-d_6_, **Fig. S17**) δ 157.7, 150.8, 138.0, 137.1, 131.5, 129.6, 128.5, 127.9, 127.72, 126.1, 125.4, 115.2, 111.4, 103.6, 96.8, 69.3. | HRMS (ESI, **Fig. S18**): m/z calculated for C_21_H_17_NO [M+H]^+^ 316.1332, found 316.1333. |
| **AC1610F1 (11)** | Step 1: 2-(4-ethoxyphenyl)-1H-pyrrolo[2,3-b]pyridine (2 g, 8.4 mmol) was suspended in a mixture of EtOAc (10 ml) and hexane (40 ml) under argon and cooled to 0°C. m-CPBA (2.7 g, 12.6 mmol, 1.5 eq) was added in portion, the reaction was slowly warmed to RT and stirred for 16h. The solvent was removed under vacuum, the residue was suspended in saturated K_2_CO_3_ solution (50 ml) and stirred vigorously for 30 min. The resulting solid was filtered and washed with water to obtain the corresponding N-oxide as a yellow solid that was dried under vacuum and used without further purification (1.5 g, 70%).  ^1^H-NMR (300 MHz, DMSO-d_6_): δ 1.35 (t, J= 7.1 Hz, 3H), 4.08 (q, J= 7.1 Hz, 2H), 6.92 (s, 1H), 6.99-7.09 (m, 3H), 7.56 (d, J= 7.1 Hz, 1H), 7.96 (d, J= 8.8 Hz, 2H), 8.08 (d, J= 6.3 Hz, 1H), 12.7 (bs, 1H).  Step 2: 2-(4-ethoxyphenyl)-1H-pyrrolo[2,3-b]pyridine 7-oxide (500 mg, 1.96 mmol) was placed under argon in a previously dried 25 ml pressure tube and dry acetonitrile (8 ml) was added, followed by dimethyl sulfate (205 µL, 2.16 mmol, 1.1 eq). The mixture was heated to 60°C for 16h. After complete formation of the O-methylated intermediate, the reaction was cooled to RT, 7M ammonia in methanol (1.4 ml, 9.8 mmol, 5 eq) was added and the tube was heated to 60°C for 16h. After cooling to RT, the mixture was filtered and the precipitate was washed with MeCN and dried under vacuum to obtain a yellow solid (270 mg, 54%). | ^1^H-NMR (300 MHz, DMSO-d_6_, **Fig. S19**): δ 11.24 (s, 1H), 7.69 (d, *J* = 8.4 Hz, 2H), 7.51 (d, *J* = 8.3 Hz, 1H), 6.93 (d, *J* = 8.4 Hz, 2H), 6.50 (d, *J* = 2.2 Hz, 1H), 6.25 (d, *J* = 8.3 Hz, 1H), 5.62 (s, 2H), 4.04 (q, *J* = 6.9 Hz, 2H), 1.34 (t, *J* = 6.9 Hz, 3H) | ^13^C NMR (75 MHz, DMSO-d_6_, **Fig. S20**): δ 157.3, 155.8, 148.9, 132.8, 129.3, 125.5, 125.3, 114.6, 112.2, 103.0, 96.1, 63.0, 14.71. | HRMS (ESI, **Fig. S21**): m/z calculated for C_15_H_15_N_3_O [M+H]^+^ 254.1288, found 254.1286. |

**Table S2 General procedures for synthesis of sibiriline-Me, sibiriline-f-Me, AC1535, AC1584 and AC1610F1.** The data related to ^1^H and ^13^C NMR and HRMS analysis are reported in the table.

**Fig. S7 ^1^H NMR spectrum of sibiriline-Me (7) in DMSO-d_6_.**

**Fig. S8 ^13^C NMR spectrum of sibiriline-Me (7) in DMSO-d_6._**


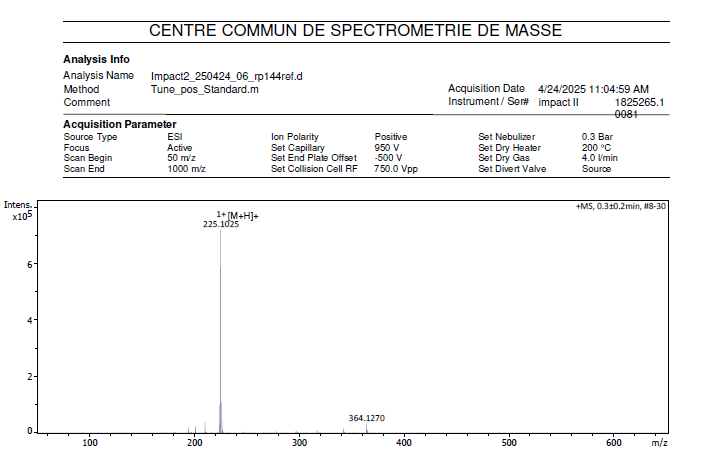


**Fig. S9 HRMS (ESI) analysis of sibiriline-Me (7).**

**Fig. S10 ^1^H NMR spectrum of sibiriline-f-Me (8) in DMSO-d_6_.**

**Fig. S11 ^13^C NMR spectrum of sibiriline-f-Me (8) in DMSO-d_6_.**


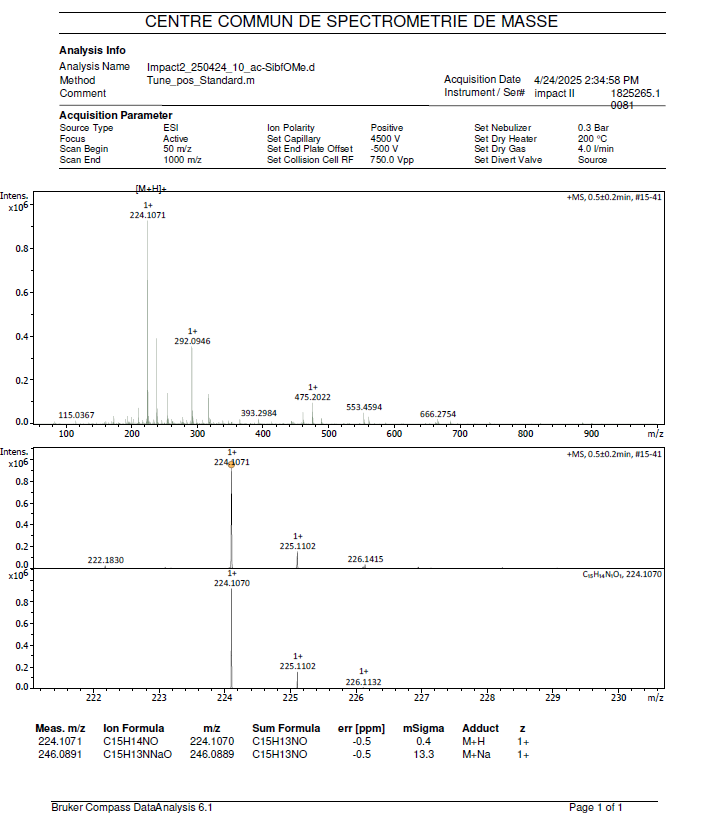


**Fig. S12 HRMS (ESI) analysis of sibiriline-f-Me (8).**

**Fig. S13 ^1^H NMR spectrum of AC1535 (9) in CDCl_3_.**

**Fig. S14 ^13^C NMR spectrum of AC1535 (9) in CDCl_3_.**


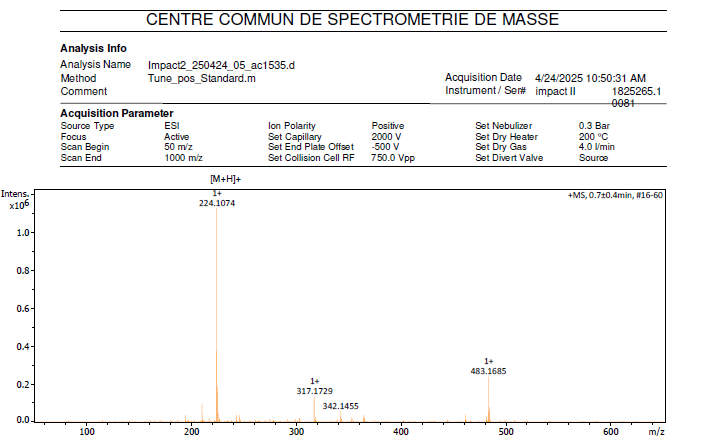


**Fig. S15 HRMS (ESI) analysis of AC1535 (9).**

**Fig. S16 ^1^H NMR spectrum of AC1584 (10) in DMSO-d_6_.**

**Fig. S17 ^13^C NMR spectrum of AC1584 (10) in DMSO-d_6_.**


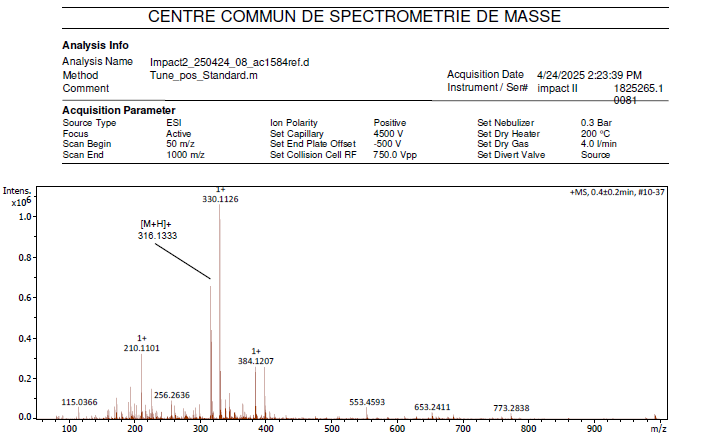


**Fig. S18 HRMS (ESI) analysis of AC1584 (10).**

**Fig. S19 ^1^H NMR spectrum of AC1610F1 (11) in DMSO-d_6_.**

**Fig. S20 ^13^C NMR spectrum of AC1610F1 (11) in DMSO-d_6_.**


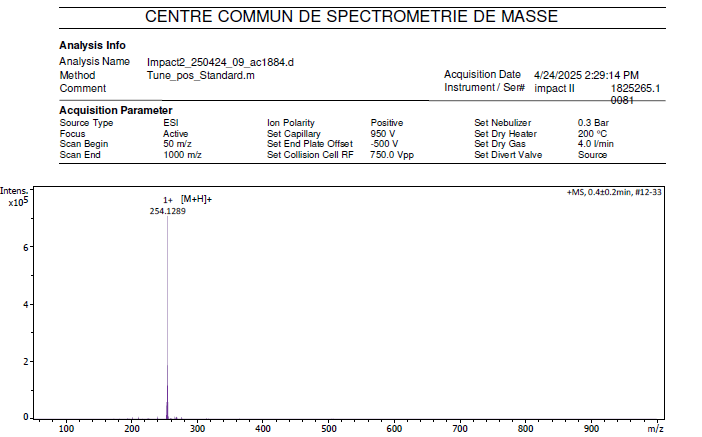


**Fig. S21 HRMS (ESI) analysis of AC1610F1 (11).**
